# ﻿Additional fauna of *Coelostoma* Brullé, 1835 from China, with re-establishment of *Coelostomasulcatum* Pu, 1963 as a valid species (Coleoptera, Hydrophilidae, Sphaeridiinae)

**DOI:** 10.3897/zookeys.1091.79564

**Published:** 2022-03-31

**Authors:** Zuqi Mai, Jian Hu, Fenglong Jia

**Affiliations:** 1 School of Agriculture, Sun Yat-sen University, Guangzhou, 511436, Guangdong, China Sun Yat-sen University Guangzhou China; 2 Institute of Entomology, Life Sciences School, Sun Yat-sen University, Guangzhou, 510275, Guangdong, China Sun Yat-sen University Guangzhou China

**Keywords:** habitat, modified key, new records, new species, valid name, variations

## Abstract

Six new species of *Coelostoma* Brullé, 1835 are described from China: *Coelostomabannanicum* Mai & Jia, **sp. nov.**, *C.dactylopunctum* Mai & Jia, **sp. nov.**, *C.fortunum* Mai & Jia, **sp. nov.**, and *C.pseudomartensi* Mai & Jia, **sp. nov.** from Yunnan; *C.mixtum* Mai & Jia, **sp. nov.** from Fujian; and *C.nankunshanense* Mai & Jia, **sp. nov.** from Guangdong. *Coelostomasurkhetensis* Hebauer, 2002 is a new record from China (Xizang). *Coelostomahuangi* Jia, Aston & Fikáček, 2014 is reported from Yunnan, *C.hajeki* Jia, Aston & Fikáček, 2014 from Hunan, *C.jaechi* Jia, Lin, Chan, Skale & Fikáček, 2017 from Guangdong, *C.turnai* Hebauer, 2006 from Fujian, Guizhou and Chongqing, and *C.wui* Orchymont, 1940 from Shanxi and Zhejiang, all for the first time. *Coelostomatranscaspicum* Reitter, 1906 is excluded from Chinese fauna. *Coelostomasulcatum* Pu, 1963 is confirmed as a valid species and its variations of aedeagus are illustrated. The specimens treated as *C.wui* Orchymont, 1940 by previous authors possibly contain two species. The diversity and habitats of Chinese *Coelostoma* are discussed. A modified key to the species of Chinese *Coelostoma* is provided.

## ﻿Introduction

*Coelostoma* Brullé, 1835 is a typical Old World group, with most of the species distributed in the Oriental, Afrotropical, and Palearctic regions. Only a few species occur in the Australian region (Hansen 1999; Jia et al. 2014; Fikáček et al. 2019). With more than 110 described species, *Coelostoma* represents one of the largest genera of Hydrophilidae. They occur in wet places with grass or stones, such as the edges of ponds and streams with grass, muddy lands beside paddy fields, under grass roots on wet stones, and the stone walls with running waters etc. Occasionally, some species can be found in small puddles. Adults usually are active at night and exhibit phototaxis (Hansen 1999; Jia 2005; Short and Hebauer 2006; Short and Fikáček 2011; Jia et al. 2014, 2017, 2019; Liu et al. 2020; Sheth et al. 2020). In the Nearctic and Neotropical regions, *Coelostoma* is replaced by the genera *Phaenonotum* Sharp, 1882, *Phaenostoma* Orchymont, 1937 and *Lachnodacnum* Orchymont, 1937 (Gustafson and Short 2010; Deler-Hernández et al. 2013; Clarkson et al. 2014; Jia et al. 2014).

*Coelostoma* can be separated from other genera of Coelostomatini by following characters combined: broadly oval and convex body shape; loosely segmented antennal club; prosternum convex medially; mesoventrite with an elevated arrowhead-shaped process in the middle; metaventral process projecting anteriorly between mesocoxae, abutting mesoventral elevation; the first metatarsomere distinctly longer than the second one; elytra with dense punctures and sutural stria, without serial punctures in most species; the first abdominal ventrite with or without carina medially (e.g., Jia et al. 2014; Sheth et al. 2020).

A total of 24 species has been recorded from China since 1874 (Sharp 1874; Orchymont 1925, 1936, 1940; Wu 1937; Pu 1963; Hebauer 2006; Jia et al. 2014, 2016, 2017, 2019; Liu et al. 2020). Among these species, *Coelostomatranscaspicum* Reitter, 1906 reported by Orchymont (1925) from Shandong is doubtful (Balfour-Browne 1951). Sheth et al. (2020) removed *C.sulcatum* Pu, 1963 from the synonymy of *C.stultum* (Walker, 1858) and considered it as a likely synonym of *C.bhutanicum* Jayaswal. Hence the status of *C.sulcatum* Pu, 1963 is now unclear.

The aim of this study is to describe the new species, update the species of the Chinese fauna, and verify the status of *C.sulcatum* Pu, 1963 as well as promoting the knowledge of Chinese *Coelostoma*. Until now, a total of 30 species has been recorded in China including the six new species in this paper.

## ﻿Material and methods

Representative specimens were dissected. After 8 min in 10% KOH at 100 °C, dissected male genitalia were transferred to a drop of distilled water, remaining membranes were removed under a compound microscope, and the cleaned genitalia were subsequently mounted in a drop of soluble resin on a piece of paper card attached below the respective specimen after photography. Habitus photographs were taken using a Nikon DS-Ri2 mounted on a Nikon SMZ25; layers were captured and stacked in the NIS-Elements software. Photographs of genitalia were taken using a Zeiss AxioCam HRc mounted on a Zeiss AX10 microscope with the Axio Vision SE64 software. These images were then stacked in Helicon focus (v7.0.2). Habitat images were taken using Canon or Nikon digital camera. SEM photographs were taken with a Phenom Pro scanning electronic microscope. All images were digitally enhanced using Adobe Photoshop CS6. Label data of the type specimens are cited verbatim and enclosed in double quotes; a slash divides separate rows on the same label, a double slash divides separate labels. Morphological terminology used in the description largely follows Hansen (1991) and Jia et al. (2014). Examined specimens are deposited in the following collections:

**HBUM**Hebei University Museum, Baoding, Hebei Province, China;

**IZCAS**Chinese Academy of Sciences, Institute of Zoology, Beijing, China;

**SYSU**Sun Yat-sen University, Guangzhou, China.

The following additional specimen was examined for comparative purposes:

*Dactylosternumlatum* (Sharp, 1873): 1 male (SYSU), China, Yunnan, Baoshan Prefecture, Gaoligong Natural Park, 24.91°N, 98.81°E, 1751 m, 22.v.2016, Yudan Tang & Ruijuan Zhang leg.

## ﻿Results

### ﻿Descriptions of new species

#### ﻿ Coelostoma (Lachnocoelostoma) bannanicum

Taxon classificationAnimaliaColeopteraHydrophilidae

Mai & Jia sp. nov.

D153350D-60A8-5CF0-9E85-B6C81EA83E84

http://zoobank.org/121BDE4F-418D-4B74-A774-8A0796B528E9

Figure 1A–E

##### Type material examined.

***Holotype***: male (SYSU), China, Yunnan, Xishuangbanna Dai Autonomous Prefecture, Mengla County, Menglun Reservoir,21°55'57"N, 101°11'25"E, 710 m, 5.vii.2016, Jiang, Liu, Huang & Liu leg. ***Paratypes***: 4 spec. (SYSU), same data as the holotype.

##### Diagnosis.

**Figure 1.**
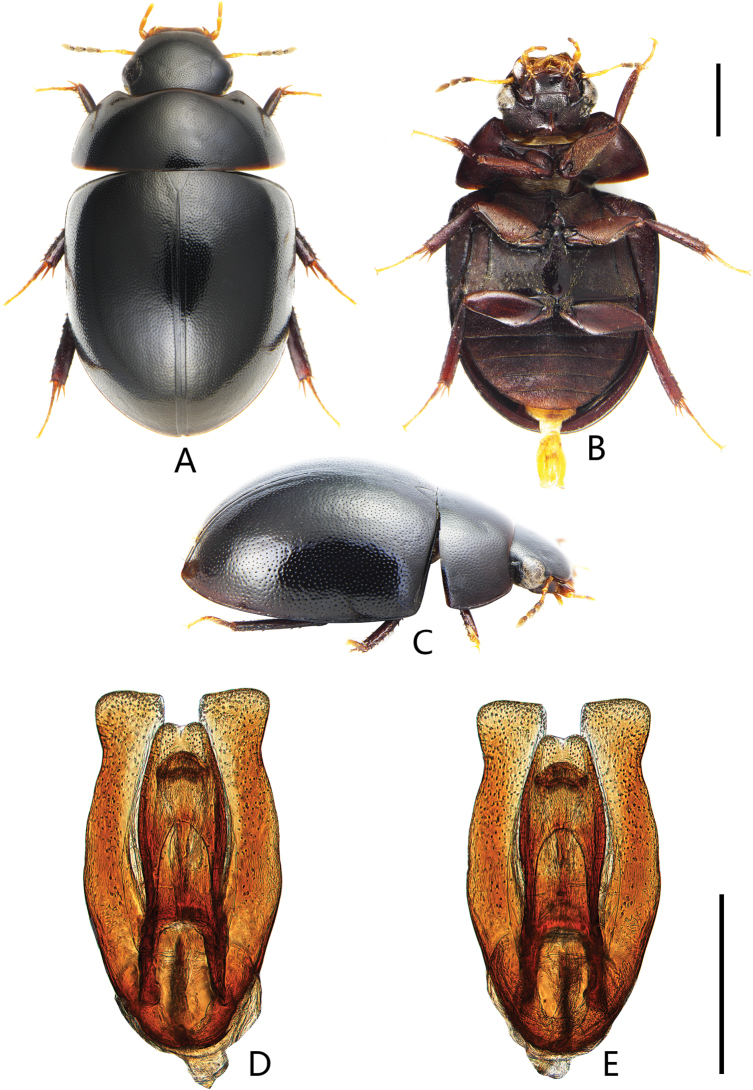
Coelostoma (Lachnocoelostoma) bannanicum Mai & Jia, sp. nov. **A** dorsal view **B** ventral view **C** lateral view **D, E** aedeagus (**D** dorsal view **E** ventral view). Scale bars :1.0 mm (**A–C**); 0.5 mm (**D, E**).

Length 5.4–5.7 mm. Head, pronotum and elytra with similar punctation. Prosternal carina with a prominent tooth anteromedially. Elytra slightly parallel-sided in the middle, without serial punctures laterally. Mesofemora densely pubescent, except on extreme apex. First abdominal ventrite with distinct median carina on basal two-thirds. Fifth ventrite slightly emarginate and with a row of stout setae apically. ***Aedeagus*** (Fig. 1D, E): 1.1 mm long. Median lobe slightly emarginated apically, slightly widened in the middle; gonopore situated subapically. Parameres longer than median lobe, strongly expanded and truncate at apex, outer apical angle rounded and inner apical angle nearly rectangular; apex of parameres wider than apex of median lobe.

##### Description.

***Form and colour*** (Fig. 1A–C). Total length 5.4–5.7 mm (holotype: 5.6 mm); maximum width 3.1–3.3 mm (holotype: 3.2 mm); body broadly oval, slightly parallel-sided in the middle, moderately convex. Dorsum black and shiny. Labrum, maxillary palpi and labial palpi reddish brown, antennae yellowish to reddish brown with dark club. Ventral surface reddish brown to black. Femora and tibiae dark reddish brown, tarsi pale reddish.

***Head*.** Dorsal surface with dense fine punctures. Interstices between punctures smooth. Clypeus subtruncate anteriorly. Eyes of moderate size, distinctly emarginated anteriorly in lateral view, separated by ca. 3.6 × the width of one eye. Mentum strongly emarginate anteriorly and depressed in anterior half, with sparse punctures except on the depressed portion. Antennae with 9 antennomeres, antennal club (antennomeres 7–9) densely pubescent. Maxillary palpomere 2 strongly swollen, palpomere 4 truncate apically, slightly longer than palpomere 3. Gula narrow and glabrous.

***Thorax*.** Pronotum widest posteriorly, gradually narrowed anteriad, with punctures as on head, anterolateral angles obtusely rounded, posterolateral angles blunt, anterior and lateral margins with narrow marginal bead. Prosternum with a carina medially and a prominent tooth anteromedially. Scutellum almost in shape of equilateral triangle, with punctures finer than those on pronotum. Elytra with more or less coarser punctures than those on pronotum, punctures on lateral and posterior portions somewhat coarser than those on disc; elytra without serial punctures; sutural stria reaching anterior third of elytra; lateral margin of elytra with bead but not explanate.

***Legs*.** Pro- and mesofemora bearing dense pubescence, except on extreme apex. Metafemora not pubescent, with dense microsculptures and spares fine punctures. Meso- and Metatibiae slightly flattened, with strong apical spurs and series of sparse stout spines laterally. Tarsi with long dorsal setae and gold ventral setae; metatarsi with fifth tarsomere almost as long as third and fourth combined. Claws curved, with a pair of long setae beneath.

***Abdomen*.** Abdominal ventrites densely pubescent. First ventrite with distinct median carina on basal two-thirds. Fifth ventrite with fine marginal bead and slightly emarginated apically, with a row of stout setae apically.

***Male genitalia*** (Fig. 1D, E). Aedeagus ca. 1.1 mm long. Median lobe slightly emarginated apically, widest in the middle, ca. 3.2 × as long as wide. Gonopore situated subapically, wider than long. Parameres longer than median lobe; outer face strongly sinuate at anterior third, strongly expanded and truncate at apex, outer apical angle rounded and inner apical angle nearly rectangular; apex of parameres wider than apex of median lobe.

##### Etymology.

This species is named after the type locality, Xishuangbanna Dai Autonomous Prefecture.

##### Biology.

On the basis of private communication to the collector, Dr. Ri-Xin Jiang (Guizhou University), the specimens were collected under stones at the edges of a mountain stream.

##### Remarks.

This species is very similar to *C.coomani* Orchymont, 1932 and *C.jaechi* Jia, Lin, Chan, Skale & Fikáček, 2017 in the weakly emarginate apex of the median lobe, and to *C.surkhetensis* Hebauer, 2002 in the shape of the median lobe. It can be distinguished from *C.coomani* (Jia et al. 2014: fig. 24) by the gonopore situated subapically (Fig. 1D, E) (situated at midlength in *C.coomani*) and outer margin of parameres strongly sinuate subapically (Fig. 1D, E) (not or only slightly sinuate in *C.coomani*). It differs from *C.jaechi* (Jia et al. 2017: fig. 11) by larger body size (body size < 5 mm in *C.jaechi*), median lobe widest at midlength (Fig. 1D, E) (widest in apical third in *C.jaechi*). It can be distinguished from *C.surkhetensis* (Fig. 8A–E) by parameres strongly expanded apically (Fig. 1D, E) (apex not expended in *C.surkhetensis* (Fig. 8D, E)), median lobe widest at midlength (Fig. 1D, E) (outer face subparallel throughout in *C.surkhetensis* (Fig. 8D, E)).

##### Distribution.

Only known from type locality. China (Yunnan).

#### ﻿ Coelostoma (Lachnocoelostoma) dactylopunctum

Taxon classificationAnimaliaColeopteraHydrophilidae

Mai & Jia sp. nov.

74C3F777-C7B1-548A-8CDA-D15A392E60F9

http://zoobank.org/4D897D7D-B3BD-48F1-A916-D47B042F9D1D

Figures 2A–E , 12A

##### Type material examined.

***Holotype***: male (SYSU), China, Yunnan, Honghe Hani and Yi Autonomous Prefecture, Lvchun County, Huanglianshan Mountain, Huanglianshan Reservoir (黄连山水库), 22.8898°N, 102.2952°E, 1717.3 m, 30.iv.2021, in a forest stream at night, Jiang, Yang, Huang & Mai leg. ***Paratypes***: 2 males (SYSU), same data as the holotype.

**Figure 2.**
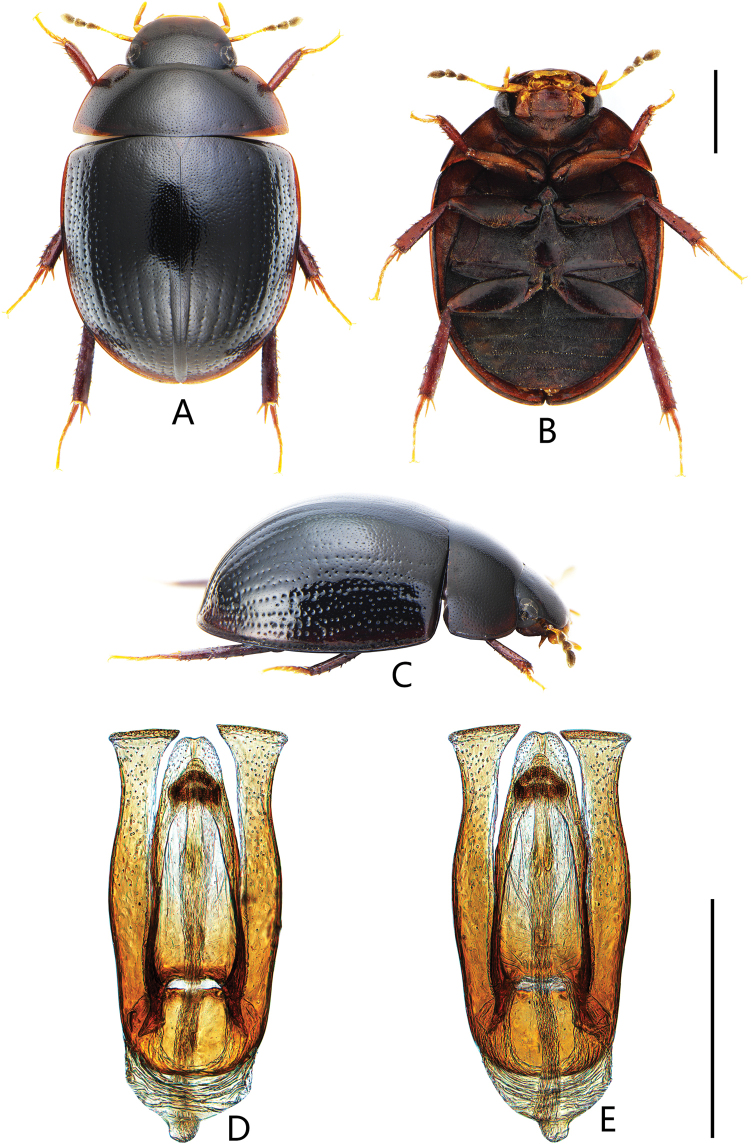
Coelostoma (Lachnocoelostoma) dactylopunctum Mai & Jia, sp. nov. **A** dorsal view **B** ventral view **C** lateral view **D, E** aedeagus (**D** dorsal view **E** ventral view). Scale bars: 1.0 mm (**A–C**); 0.5 mm (**D, E**).

##### Diagnosis.

Length 4.3–4.5 mm. Head and pronotum with similar punctation. Prosternum carinate medially, with a fine tooth anteromedially. Elytra not parallel-sided in the middle, each elytron with ten distinct rows of serial punctures; intervals between series with two sizes of punctures especially in posterior half of elytron, the finer punctures as on pronotum and much finer and shallower than the coarser punctures, coarser punctures almost as coarse as those of the series (Fig. 12A). Mesofemora densely pubescent, except on extreme apex. First abdominal ventrite with complete median carina. Fifth ventrite slightly emarginate and with a row of stout setae apically. ***Aedeagus*** (Fig. 2D, E): 0.87 mm long. Median lobe emarginate apically, outer face nearly parallel-sided from basal to middle, then gradually narrowing towards apex, gonopore situated subapically, wider than long. Parameres slightly longer than median lobe, gradually expanded from anterior fourth to apex, broadly truncate apically, inner apical angle acute, pointed; middle part of parameres ca. 0.5 × as wide as median lobe at the same level.

##### Description.

***Form and colour*** (Fig. 2D, E). Total length 4.3–4.5 mm (holotype: 4.5 mm); maximum width 2.8–3.0 mm (holotype: 3.0 mm); body broadly oval, not parallel-sided in the middle, moderately convex. Dorsum black to reddish brown, with lateral margin of pronotum and elytra paler. Labrum, maxillary palpi and labial palpi reddish brown, antennae yellowish to reddish brown with dark club. Ventral surface reddish brown. Femora and tibiae dark reddish brown, tarsi pale reddish.

***Head*.** Dorsal surface with dense fine punctures. Interstices between punctures smooth. Clypeus subtruncate anteriorly. Eyes of moderate size, slightly emarginated anteriorly in lateral view, separated by ca. 5.3 × the width of one eye. Mentum emarginate anteriorly and depressed in anterior half, with sparse punctures except on the depressed portion. Antennae with nine antennomeres, antennal club (antennomeres 7–9) densely pubescent. Maxillary palpomere 2 strongly swollen, palpomere 4 truncate apically, slightly longer than palpomere 3. Gula narrow and glabrous.

***Thorax*.** Pronotum widest posteriorly, gradually narrowed anteriad, with punctures as on head, anterolateral angles obtusely rounded, posterolateral angles blunt, anterior and lateral margins with narrow marginal bead. Prosternum with a carina medially and slightly projecting anteromedially. Scutellum in shape of equilateral triangle, with punctures as on pronotum. Each elytron with ten distinct rows of serial punctures; intervals between series with two sizes of punctures especially in posterior half of elytron, the finer punctures as on pronotum and much finer and shallower than the coarser punctures, coarser punctures almost as coarse as those of the series (Fig. 12A); series 1 and 2 reaching basal half of elytron, series 1 overlap with sutural stria; series 3 and 4 nearly reaching elytral base and fused together subposteriorly; series 5–10 reaching elytral base, slightly sulcate posteriorly; the outer four serial punctures coarser and stronger than the inner six serial punctures (Fig. 12A). Lateral margin of elytra with bead and slightly explanate.

***Legs*.** Pro- and mesofemora bearing dense pubescence, except on extreme apex. Metafemora not pubescent, with dense microsculptures and spares fine punctures. Meso- and Metatibia slightly flattened, with strong apical spurs and series of sparse stout spines laterally. Tarsi with long dorsal setae and gold ventral setae; metatarsi with fifth tarsomere almost as long as third and fourth combined. Claws curved, with a pair of long setae beneath.

***Abdomen*.** Abdominal ventrites densely pubescent. First ventrite with complete median carina. Fifth ventrite slightly emarginate and with fine marginal bead, with a row of stout setae apically.

***Male genitalia*** (Fig. 2D, E). Aedeagus ca. 0.87 mm long. Median lobe emarginate apically, widest basally, ca. 3.5 × as long as wide; outer face nearly parallel-sided from base to middle, then gradually narrowing towards apex; gonopore situated subapically, wider than long. Parameres slightly longer than median lobe, gradually expanded from anterior fourth to apex, broadly truncate apically, inner apical angle acute, pointed; middle part of parameres ca. 0.5 × as wide as median lobe at the same level.

##### Etymology.

The species name is a combination of *Dactylosternum*, a genus in the same tribe Coelostomatini, and the Latin *punctum*. The name refers to the fact that this species with distinct serial punctures on elytra which is similar to many species of *Dactylosternum* Wollaston, 1854.

##### Biology.

Aquatic. Adults were found on the edges of a forest stream at night.

##### Remarks.

This species is easily recognized as a member of *Dactylosternum* by the ten rows of serial punctures on elytra, which is different to any known *Coelostoma* species. *Coelostomamartensi* Hebauer, 2002 and *C.gentilii* Jia, Aston & Fikáček, 2014 (Fig. 13B) are also known with serial punctures on elytra, but only visible laterally. *Coelostomarubens* Hebauer, 2002 (Jia et al. 2019: figs 4, 5), *C.jaculum* Jia, Angus & Bian, 2019 (Jia et al. 2019: figs 2, 3) and *C.phototropicum* Jia, Angus & Bian (Jia et al. 2019: fig. 1) are similar to this new species in the shape of aedeagus. However, none of these species with serial punctures on elytra.

##### Distribution.

Only known from type locality. China (Yunnan).

#### ﻿ Coelostoma (Lachnocoelostoma) fortunum

Taxon classificationAnimaliaColeopteraHydrophilidae

Mai & Jia sp. nov.

34306785-FB62-558E-BA28-CC52CE49EEB6

http://zoobank.org/878E3932-8BA5-44B0-8F9F-B255EB1A0268

Figures 3A–E , 12B

##### Type material examined.

***Holotype***: male (SYSU), China, Yunnan, Dehong Dai and Jingpo Autonomous Prefecture, Yingjiang County, Xima Town (昔马镇), Hulukou (葫芦口), Xingyun Secondary power station (星云二级电站), 1000 m, vi.2019, light trap, Zhao-yang Tang leg. ***Paratypes***: 2 females (SYSU), same data as the holotype.

##### Diagnosis.

**Figure 3.**
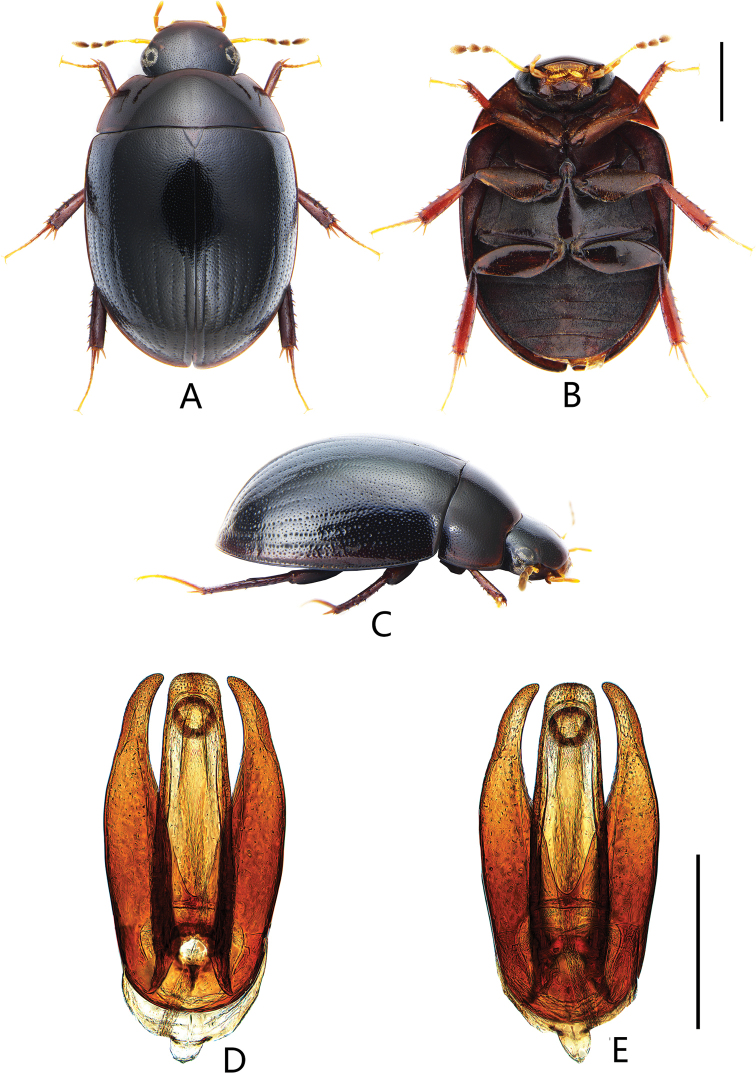
Coelostoma (Lachnocoelostoma) fortunum Mai & Jia, sp. nov. **A** dorsal view **B** ventral view **C** lateral view **D, E** aedeagus (**D** dorsal view **E** ventral view). Scale bars: 1.0 mm (**A–C**); 0.5 mm (**D, E**).

Length 4.4–4.6 mm. Head and pronotum with similar punctation. Prosternum carinate medially, with a prominent tooth anteromedially. Elytra not parallel-sided in the middle, each elytron with 10 distinct rows of serial punctures; intervals between series with two sizes of punctures especially in posterior half of elytron, all finer than those of the series, the finer punctures finer and shallower than the coarser punctures but not extremely so (Fig. 12B); lateral margin of elytra with bead but not explanate. Mesofemora densely pubescent, except on extreme apex. First abdominal ventrite with carina on basal two-thirds. Fifth ventrite slightly emarginate and with a row of stout setae apically. ***Aedeagus*** (Fig. 3D, E): 1.1 mm long. Median lobe widest basally, almost truncate apically, outer face nearly parallel-sided throughout; gonopore situated subapically, rounded, almost as wide as long. Parameres almost the same length as median lobe, outer face continually curved, apex of paramere pointed, curved inwards.

##### Description.

***Form and colour*** (Fig. 3A–C). Total length 4.4–4.6 mm (holotype: 4.5 mm); maximum width 2.6–2.7 mm (holotype: 2.7 mm); body broadly oval, not parallel-sided in the middle, moderately convex. Dorsum black to reddish brown, with lateral margin of pronotum and elytra slightly paler. Labrum, maxillary palpi and labial palpi reddish brown, antennae yellowish to reddish brown with dark club. Ventral surface reddish brown. Femora and tibiae dark reddish brown, tarsi pale reddish.

***Head*.** Dorsal surface with dense fine punctures. Interstices between punctures smooth. Clypeus subtruncate anteriorly. Eyes of moderate size, slightly emarginated anteriorly in lateral view, separated by ca. 4.2 × the width of one eye. Mentum emarginated anteriorly and depressed in anterior half, with sparse punctures and transverse microsculpture. Antennae with 9 antennomeres, antennal club (antennomeres 7–9) densely pubescent. Maxillary palpomere 2 strongly swollen, palpomere 4 truncate apically, slightly longer than palpomere 3. Gula narrow and glabrous.

***Thorax*.** Pronotum widest posteriorly, gradually narrowed anteriad, with punctures slightly sparser than those on head, anterolateral angles obtusely rounded, posterolateral angles blunt, anterior and lateral margins with narrow marginal bead. Prosternum with a carina medially and a prominent tooth anteromedially. Scutellum in shape of equilateral triangle, with punctures as on pronotum. Each elytron with ten distinct rows of serial punctures; intervals between series with two sizes of punctures especially in posterior half of elytron, all finer than those of the series, the finer punctures finer and shallower than the coarser punctures but not extremely so (Fig. 12B); series 1–4 only visible in posterior half of elytron, series 1 overlaps with sutural stria, series 3 and 4 fused together subposteriorly; series 5–10 nearly reaching elytral base, slightly sulcate posteriorly. The outer four serial punctures coarser and stronger than the inner six serial punctures (Fig. 12B). Lateral margin of elytra with bead but not explanate.

***Legs*.** Pro- and mesofemora bearing dense pubescence, except on extreme apex. Metafemora not pubescent, with dense microsculptures and spares fine punctures. Meso- and Metatibia slightly flattened, with strong apical spurs and series of sparse stout spines laterally. Tarsi with long dorsal setae and gold ventral setae; metatarsi with fifth tarsomere almost as long as third and fourth combined. Claws curved, with a pair of long setae beneath.

***Abdomen*.** Abdominal ventrites densely pubescent. First ventrite with distinct median carina on basal two-thirds. Fifth ventrite slightly emarginate and with fine marginal bead, with a row of stout setae apically.

***Male genitalia*** (Fig. 3D, E). Aedeagus ca. 1.1 mm long. Median lobe widest basally, ca. 3.9 × as long as wide, almost truncate apically, outer face nearly parallel-sided throughout; gonopore situated subapically, rounded, almost as wide as long. Parameres almost the same length as median lobe, outer face continually curved, apex of paramere pointed, curved inwards.

##### Etymology.

This new species is derived from the Latin adjective *fortuna*, fortunate, meaning the senior author was lucky to collect the new species.

##### Biology.

All specimens were collected in a light trap

##### Remarks.

This species also with ten rows of serial punctures on elytra as *C.dactylopunctum* sp. nov. It can be distinguished from the latter by apex of median lobe truncate and not emarginate (Fig. 3D, E) (slightly emarginate and rounded apically in *C.dactylopunctum* sp. nov. (Fig. 2D, E)), apex of paramere pointed (Fig. 3D, E) (paramere broadly truncate apically in *C.dactylopunctum* sp. nov. (Fig. 2D, E)), first ventrite with median carina on basal two-thirds (with complete median carina in *C.dactylopunctum* sp. nov.).

##### Distribution.

Only known from type locality. China (Yunnan).

#### ﻿ Coelostoma (Lachnocoelostoma) mixtum

Taxon classificationAnimaliaColeopteraHydrophilidae

Mai & Jia sp. nov.

A71FE8B1-89D1-5971-8CA3-8299629BD9EF

http://zoobank.org/DBD0A5A3-6C16-435E-B4EF-463D3632DF6F

Figures 4A–C , 5A–C

##### Type material examined.

***Holotype***: male (SYSU), China, Fujian, Wuyishan, Sangang Village (三港村), 16–28.v.2004, Cai-xia Yuan & Jing Li leg.

##### Diagnosis.

Length 6.13 mm. Head, pronotum and elytra with similar punctation. Prosternum carinate medially, with a prominent tooth anteromedially. Elytra parallel-sided in the middle, without serial punctures laterally. Mesofemora densely pubescent, except on extreme apex. First abdominal ventrite with median carina on basal one-thirds. Fifth ventrite slightly emarginate and with a row of stout setae apically. ***Aedeagus*** (Fig. 5A–C): very large, similar to *Coelostomavagum* Orchymont, 1940, but median lobe narrowly rounded apically, apex without a sharp prominent tooth ventrally; parameres abruptly widened apically, distinctly bent inward.

##### Description.

**Figure 4.**
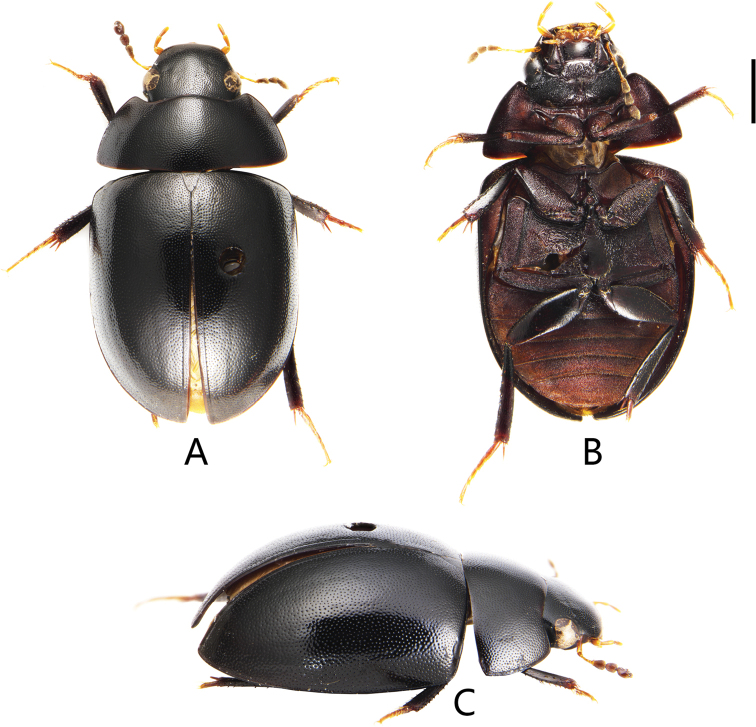
Coelostoma (Lachnocoelostoma) mixtum Mai & Jia, sp. nov. **A** dorsal view **B** ventral view **C** lateral view. Scale bar: 1.0 mm (**A–C**).

***Form and colour*** (Fig. 4A–C). Total length 6.13 mm; maximum width 3.3 mm; body broadly oval, parallel-sided in the middle, moderately convex. Dorsum black and shiny. Labrum, maxillary palpi and labial palpi reddish brown, antennae yellowish to reddish brown with dark club. Ventral surface reddish brown to black. Femora and tibiae dark reddish brown, tarsi pale reddish.

***Head*.** Dorsal surface with dense fine punctures. Interstices between punctures smooth. Clypeus subtruncate anteriorly. Eyes of moderate size, distinctly emarginate anteriorly in lateral view, separated by ca. 4 × the width of one eye. Mentum strongly emarginated anteriorly and depressed in anterior half, with sparse fine punctures. Antennae with nine antennomeres, antennal club (antennomeres 7–9) densely pubescent. Maxillary palpomere 2 strongly swollen, palpomere 4 truncate apically, slightly longer than palpomere 3. Gula narrow and glabrous.

**Figure 5.**
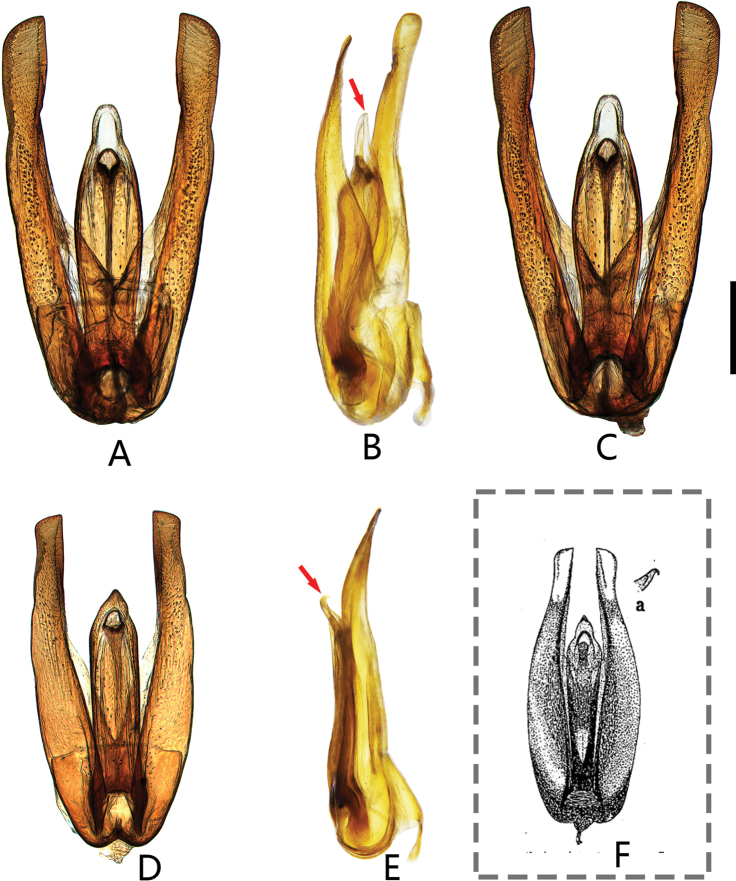
Aedeagus of *Coelostomamixtum* Mai & Jia, sp. nov. and *Coelostomavagum* Orchymont, 1940. **A–C***C.mixtum* sp. nov. **A** dorsal view **B** lateral view **C** ventral view **D–F***C.vagum***D** dorsal view **E** lateral view **F** illustration by Orchymont (1940). Scale bar: 0.5 mm (**A–E**).

***Thorax*.** Pronotum widest posteriorly, gradually narrowed anteriad, with punctures as on head, anterolateral angles obtusely rounded, posterolateral angles blunt, anterior and lateral margins with narrow marginal bead. Prosternum with a carina medially and a prominent tooth anteromedially. Scutellum in shape of equilateral triangle, with punctures finer and denser than those on pronotum. Elytra with punctures as on pronotum; elytra without serial punctures; sutural stria reaching anterior half of elytra; lateral margin of elytra with bead but not explanate.

***Legs*.** Pro- and mesofemora bearing dense pubescence, except on extreme apex. Metafemora not pubescent, with dense microsculptures and spares fine punctures. Meso- and Metatibia slightly flattened, with strong apical spurs and series of sparse stout spines laterally. Tarsi with long dorsal setae and gold ventral setae; metatarsi with fifth tarsomere almost as long as third and fourth combined. Claws curved, with a pair of long setae beneath.

***Abdomen*.** Abdominal ventrites densely pubescent. First ventrite with distinct median carina on basal one-thirds. Fifth ventrite with fine marginal bead and slightly emarginate apically.

***Male genitalia*** (Fig. 5A–C). Aedeagus very large, 2.3 mm long. Median lobe widest at midlength, ca. 5 × as long as wide; median lobe gradually narrowing towards apex, with a small finger like apex, narrowly rounded apically; gonopore rounded, situated subapically; parameres much longer than the median lobe, abruptly widened apically, distinctly bent inward.

##### Etymology.

This species is derived from the Latin verb *mixtus*, mix, refers to the fact that this species is similar to *Coelostomavagum* Orchymont, 1940 in shape of the median lobe and similar to *C.wui* Orchymont, 1940 in shape of parameres.

##### Biology.

Unknown, this species was collected with *C.wui* Orchymont, 1940 in the same place.

##### Remarks.

The holotype of this species was identified as *C.vagum* Orchymont, 1940 in Jia et al. (2017). However, it is different from *C.vagum* in the form of the aedeagus. Hence, the distribution of *C.vagum* in Fujian needs to be verified. This species is the seventh known species of the *Coelostomaphallicum* group. This group can be recognized by a large and very elongate aedeagus with an extremely reduced phallobase and large subapical gonopore (Liu et al. 2020). This species is most similar to *C.vagum* Orchymont, 1940 (Fig. 5D–F) and *C.bipunctatum* Jayaswal, 1972 in shape of the median lobe but can be distinguished from them by the narrowly rounded apex of median lobe (apex pointed in *C.vagum* and *C.bipunctatum*), median lobe widest in the middle (median lobe nearly parallel-sided in the middle in *C.vagum* and *C.bipunctatum*). It also can be distinguished from *C.vagum* by apex of median lobe without a sharp prominent tooth ventrally (Fig. 5B) (apex with a sharp prominent hook-shaped tooth ventrally in *C.vagum* (Fig. 5E))

##### Distribution.

Only known from type locality. China (Fujian).

#### ﻿ Coelostoma (Lachnocoelostoma) nankunshanense

Taxon classificationAnimaliaColeopteraHydrophilidae

Mai & Jia sp. nov.

7BD34D00-1843-587B-921A-3ABAB40A6723

http://zoobank.org/13D63201-5029-4548-8B86-1BD8BEBCA175

**Figure 6.**
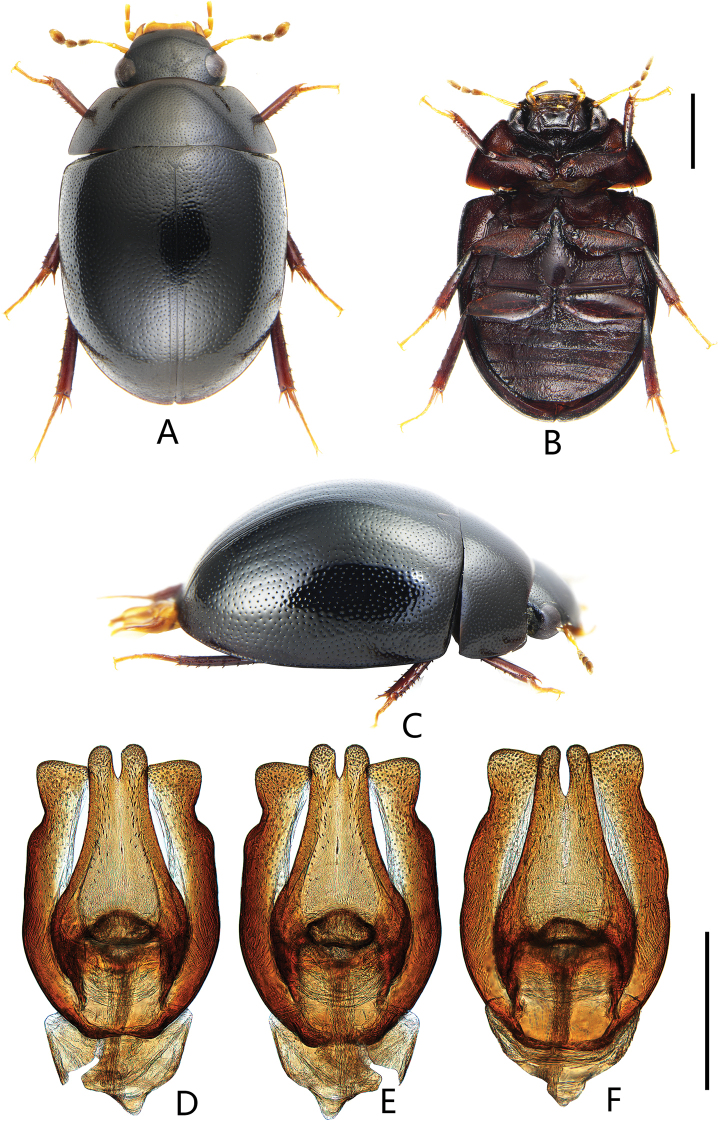
Coelostoma (Lachnocoelostoma) nankunshanense Mai & Jia, sp. nov. **A** dorsal view **B** ventral view **C** lateral view **D–F** aedeagus **D, E** holotype **D** dorsal view **E** ventral view **F** aedeagus of a paratype (dorsal view). Scale bars: 1.0 mm (**A–C**); 0.5 mm (**D–F**).

Figures 6A–F , 16G, K

##### Type material examined.

***Holotype***: male (SYSU), China, Guangdong, Longmen County, Nankunshan Mountain, Guanyintan (观音潭), 23°38'13"N, 113°51'1"E, 503.7 m, 24.iii.2021, Mai, Jiang, Yang & Huang leg. ***Paratypes***: 14 spec. (SYSU), 2 spec. (IZCAS), same data as the holotype;

##### Diagnosis.

Length 4.7–5.0 mm. Head, pronotum and elytra with similar punctation. Prosternum carinate medially, with a prominent tooth anteromedially. Elytra slightly parallel-sided in the middle, without serial punctures laterally. Mesofemora densely pubescent, except on extreme apex. First abdominal ventrite with median carina on basal two-thirds. Fifth ventrite slightly emarginate and with a row of stout setae apically. ***Aedeagus*** (Fig. 6D–F): similar to *Coelostomaturnai* Hebauer, 2006 (Jia et al. 2014: fig. 21), but median lobe wider and shorter, outer face of parameres distinctly incised subapically.

##### Description.

***Form and colour*** (Fig. 6A–C). Total length 4.7–5.0 mm (holotype: 4.9 mm); maximum width 2.6–3.2 mm (holotype: 3.0 mm); body broadly oval, slightly parallel-sided in the middle, moderately convex. Dorsum black and shiny. Labrum, maxillary palpi and labial palpi reddish brown, antennae yellowish to reddish brown with dark club. Ventral surface reddish brown to black. Femora and tibiae dark reddish brown, tarsi pale reddish.

***Head*.** Dorsal surface with dense fine punctures. Interstices between punctures smooth. Clypeus subtruncate anteriorly. Eyes of moderate size, distinctly emarginate anteriorly in lateral view, separated by ca. 3.5 × the width of one eye. Mentum strongly emarginated anteriorly and depressed in anterior half, with sparse punctures and dense transverse microsculpture. Antennae with nine antennomeres, antennal club (antennomeres 7–9) densely pubescent. Maxillary palpomere 2 strongly swollen, palpomere 4 truncate apically, slightly longer than palpomere 3. Gula narrow and glabrous.

***Thorax*.** Pronotum widest posteriorly, gradually narrowed anteriad, with punctures as on head, anterolateral angles obtusely rounded, posterolateral angles blunt, anterior and lateral margins with narrow marginal bead. Prosternum with a carina medially and a prominent tooth anteromedially. Scutellum in shape of equilateral triangle, with punctures finer and denser than those on pronotum. Elytra with punctures as on pronotum, punctures on lateral and posterior portions somewhat coarser than those on disc; elytra without serial punctures; sutural stria reaching anterior third of elytra; lateral margin of elytra with bead but not explanate.

***Legs*.** Pro- and mesofemora bearing dense pubescence, except on extreme apex. Metafemora not pubescent, with dense microsculptures and spares fine punctures. Meso- and Metatibia slightly flattened, with strong apical spurs and series of sparse stout spines laterally. Tarsi with long dorsal setae and gold ventral setae; metatarsi with fifth tarsomere almost as long as third and fourth combined. Claws curved, with a pair of long setae beneath.

***Abdomen*.** Abdominal ventrites densely pubescent. First ventrite with distinct median carina on basal two-thirds. Fifth ventrite with fine marginal bead and slightly emarginate apically.

***Male genitalia*** (Fig. 6D–F). Aedeagus ca. 1.0 mm long. Median lobe widest basally, bottle-shaped with apex emarginate (depth of emargination are variable in individuals as in Fig. 6F), ca. 1.8 × as long as wide; gonopore situated basally, wider than long; parameres slightly shorter than median lobe, distinctly incised on outer face subapically, broadly truncate apically

##### Etymology.

This species is named after the type locality, Nankunshan, a nature reserve in Guangdong Province.

##### Biology

**(Figs 16G, K).** All specimens were collected at night on some large stones in a forest stream. Individuals feed on algal mat and mate on the habitat. Some individuals of *Coelostomabifidum* Jia, Aston & Fikáček, 2014 were also collected together.

##### Distribution.

Only known from type locality. China (Guangdong).

#### ﻿ Coelostoma (Lachnocoelostoma) pseudomartensi

Taxon classificationAnimaliaColeopteraHydrophilidae

Mai & Jia sp. nov.

A05D12AA-BC58-58E0-9809-1DEFFF295E95

http://zoobank.org/3A2C7D93-1840-43BD-A0C7-B99FA4BB2DDB

Figures 7A–E , 13A

##### Type material examined.

***Holotype***: male (SYSU), China, Yunnan, Honghe Hani and Yi Autonomous Prefecture, Lvchun County, Huanglianshan Mountain, Huanglianshan Reservoir (黄连山水库), 22.8898°N, 102.2952°E, 1717.3 m, 30.iv.2021, in a forest stream at night, Jiang, Yang, Huang & Mai leg.

##### Diagnosis.

**Figure 7.**
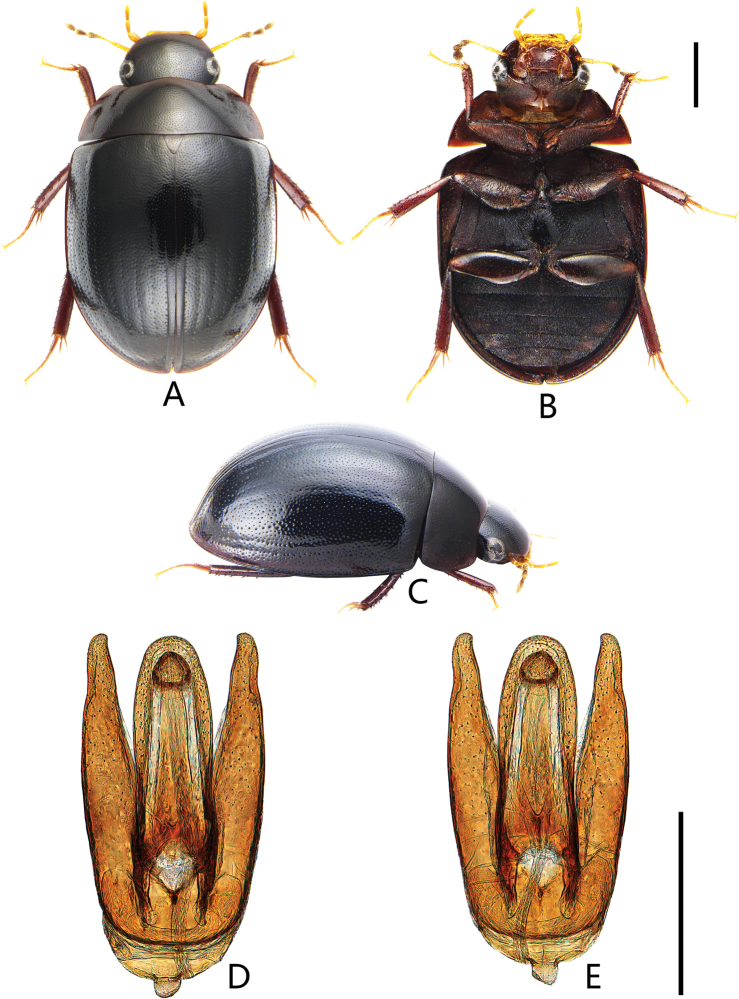
Coelostoma (Lachnocoelostoma) pseudomartensi M Jia, sp. nov. **A** dorsal view **B** ventral view **C** lateral view **D, E** aedeagus **D** dorsal view **E** ventral view. Scale bars: 1.0 mm (**A–C**); 0.5 mm (**D, E**).

Length 5.4 mm. Head and pronotum with similar punctation. Prosternum carinate medially, with a prominent tooth anteromedially. Elytra slightly parallel-sided in the middle, each elytron with ten serial punctures, somewhat difficult to separate them from the ground punctures in anterior half of elytron; intervals between series with two sizes of punctures especially in posterior half of elytron, coarser punctures slightly finer than those of the series (Fig. 13A). lateral margin of elytra with bead but not explanate. Mesofemora densely pubescent, except on extreme apex. First abdominal ventrite with carina on basal half. Fifth ventrite slightly emarginate and with a row of stout setae apically. ***Aedeagus*** (Fig. 7D, E): 1.0 mm long. Median lobe widest at anterior third, rounded apically, outer face nearly parallel-sided in the middle; gonopore situated subapically, in shape of triangle, almost as wide as long. Parameres almost the same length as median lobe, straight, not curved, gradually narrowed from middle to apex, narrowly rounded apically.

##### Description.

***Form and color*** (Fig. 7A–C). Total length 5.4 mm; maximum width 3.3 mm; body broadly oval, nearly parallel-sided in the middle, moderately convex. Dorsum black, with lateral margin of pronotum and elytra dark reddish brown. Labrum, maxillary palpi and labial palpi reddish brown, antennae yellowish to reddish brown with dark club. Ventral surface reddish brown. Femora and tibiae dark reddish brown, tarsi pale reddish.

***Head*.** Dorsal surface with dense fine punctures. Interstices between punctures smooth. Clypeus subtruncate anteriorly. Eyes of moderate size, slightly emarginate anteriorly in lateral view, separated by ca. 4.5 × the width of one eye. Mentum strongly emarginate anteriorly and depressed in anterior half, with sparse punctures and transverse microsculpture. Antennae with 9 antennomeres, antennal club (antennomeres 7–9) densely pubescent. Maxillary palpomere 2 strongly swollen, palpomere 4 truncate apically, slightly longer than palpomere 3. Gula narrow and glabrous.

***Thorax*.** Pronotum widest posteriorly, gradually narrowed anteriad, with punctures as on head, anterolateral angles obtusely rounded, posterolateral angles blunt, anterior and lateral margins with narrow marginal bead. Prosternum with a carina medially and a prominent tooth anteromedially. Scutellum slightly longer than wide, in shape of equilateral triangle, with punctures as on pronotum. Elytra with ground punctures as on pronotum, becoming coarser posteriorly. Each elytron with ten rows of serial punctures, somewhat difficult to separate them from the ground punctures in anterior half of elytron; intervals between series with two sizes of punctures especially in posterior half of elytron, coarser punctures slightly finer than those of the series; series 1 overlap with sutural stria; series 8 and 9 slightly sulcate posteriorly; series 10 short and becoming indistinct posteriorly (Fig. 13A). Lateral margin of elytra with bead, not explanate.

***Legs*.** Pro- and mesofemora bearing dense pubescence, except on extreme apex. Metafemora not pubescent, with dense microsculptures and spares fine punctures. Meso- and Metatibia slightly flattened, with strong apical spurs and series of sparse stout spines laterally. Tarsi with long dorsal setae and gold ventral setae; metatarsi with fifth tarsomere almost as long as third and fourth combined. Claws curved, with a pair of long setae beneath.

***Abdomen*.** Abdominal ventrites densely pubescent. First ventrite with distinct median carina on basal half. Fifth ventrite slightly emarginate and with fine marginal bead, with a row of stout setae apically.

***Male genitalia*** (Fig. 7D, E). Aedeagus ca. 1.0 mm long. Median lobe widest at anterior third, ca. 3.7 × as long as wide, rounded apically, slightly narrowed in the middle; gonopore situated subapically, in shape of triangle, almost as wide as long. Parameres straight, widest basally, almost the same length as median lobe, not curved inwards, gradually narrowed from middle to apex, with a narrowed and rounded apex.

##### Etymology.

This species name is a combination of the Latin *pseudo*-, false, and another species of the genus, *Coelostomamartensi*. The name refers to the fact that this species is similar to *C.martensi* in some morphology characters.

##### Biology.

This species lives mixed with *Coelostomadactylopunctum* sp. nov.

##### Remarks.

This species is similar to *Coelostomamartensi* Hebauer, 2002 (Hebauer 2002: fig. 7; Jia et al. 2014: fig. 28) not only in the shape of aedeagus but also in the serial punctures on lateral elytra. It can be distinguished from the latter by median lobe widest subapically (widest medially in *C.martensi*), paramere with a narrowed and rounded apex (apex of paramere broad and truncate in *C.martensi*), elytron with ten rows of serial punctures (only with serial punctures sublaterally in *C.martensi*).

##### Distribution.

Only known from type locality. China (Yunnan).

### ﻿New records and faunistic data

#### ﻿ Coelostoma (Lachnocoelostoma) surkhetensis

Taxon classificationAnimaliaColeopteraHydrophilidae

Hebauer, 2002

42C8BD25-019C-5DDE-B6BD-FE5D9CF5AA0F

Figures 8A–E , 16H

##### Material examined.

**China: Xizang**: 1 male (SYSU), Muotuo County, Miri Village, 29°25'06"N, 95°24'23"E, 820 m, 23.vi.2018, Shi-shuai Wang & Zu-long Liang leg.; 6 spec. (SYSU), Muotuo County, Muotuo Town, 29.269°N, 95.227°E, 766 m, in a stream beside 219 national highway, Qian-le Lu leg.

##### Diagnosis.

**Figure 8.**
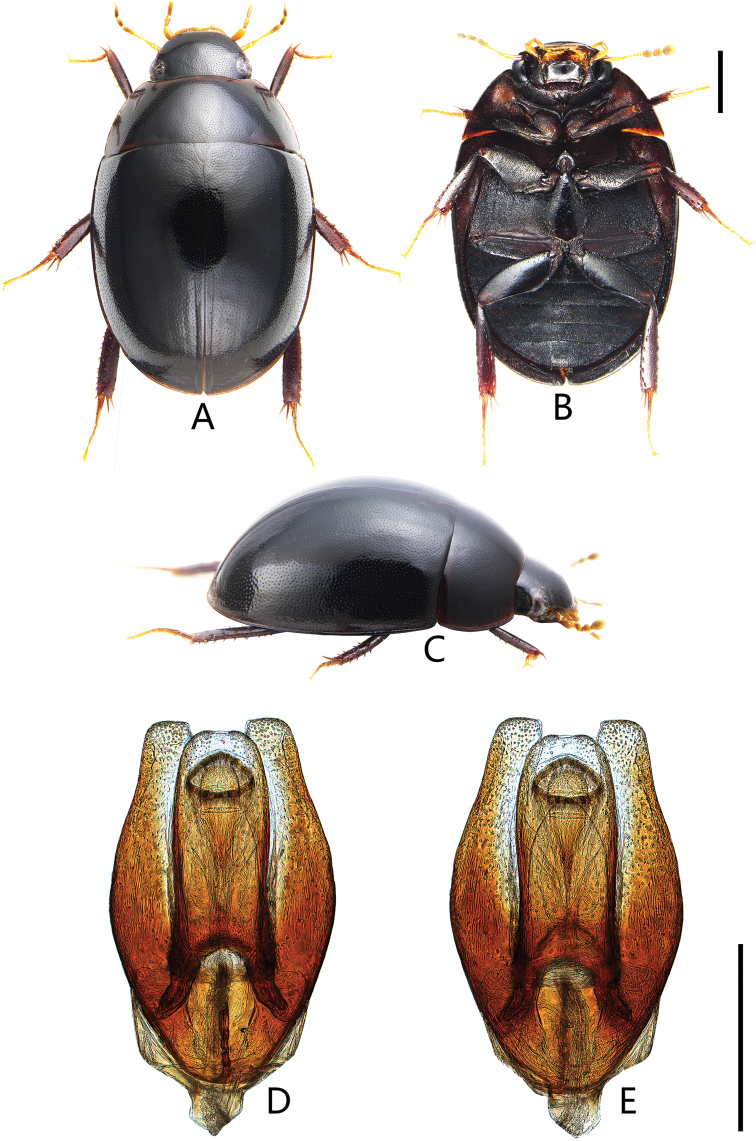
Coelostoma (Lachnocoelostoma) surkhetensis Hebauer, 2002 **A** dorsal view **B** ventral view **C** lateral view **D, E** aedeagus **D** dorsal view **E** ventral view. Scale bars: 1.0 mm (**A–C**); 0.5 mm (**D, E**).

Length 5.4–5.8 mm. Head, pronotum and elytra with similar punctation. Prosternum carinate medially, with a prominent tooth anteromedially. Elytra not parallel-sided in the middle, without serial punctures laterally. Mesofemora densely pubescent, except on extreme apex. First abdominal ventrite with distinct median carina on basal two-thirds. Fifth ventrite slightly emarginate and with a row of stout setae apically. ***Aedeagus*** (Fig. 8D, E): 1.2 mm long. Median subtruncate apically, nearly parallel-sided throughout; gonopore situated subapically. 

##### Biology

**(Fig. 16H).** On the basis of private communication to collector Mr. Qian-Le Lu, the specimens from Xizang were collected on wet stones on the edges of a forest stream. A single specimen of *C.phototropicum* Jia, Angus & Bian, 2019 was collected at the same place.

##### Distribution.

Previously known only from Nepal (Hebauer 2002; Przewoźny 2021). New record for China (Xizang).

#### ﻿ Coelostoma (Lachnocoelostoma) huangi

Taxon classificationAnimaliaColeopteraHydrophilidae

Jia, Aston & Fikáček, 2014

E891A01F-EFDA-5DD8-BDB8-A58423A7AE1E

##### Material examined.

**China: Yunnan**: 32 spec. (SYSU), Qvjing, Shizong (师宗) County, Fenghuanggu (凤凰谷) Valley, 24.618168°N, 104.264414°E, 929 m, 20.v.2021, Bao-ping Huang, Zhuo-yin Jiang & Zu-qi Mai leg.

##### Biology.

In Yunnan, specimens were collected along sides of a rocky stream in a valley at night. *Coelostomacoomani* Orchymont, 1932 and *C.phallicum* Orchymont, 1940 were also collected in the same habitat. Adult individuals attracted by light (Jia et al. 2014). Parameres slightly longer than median lobe, gradually broadened from base to middle, outer face not curved, apical third parallel-sided, apex narrower than apex of median lobe, truncate, rounded outwards and nearly rectangularly inwards.

**Figure 9.**
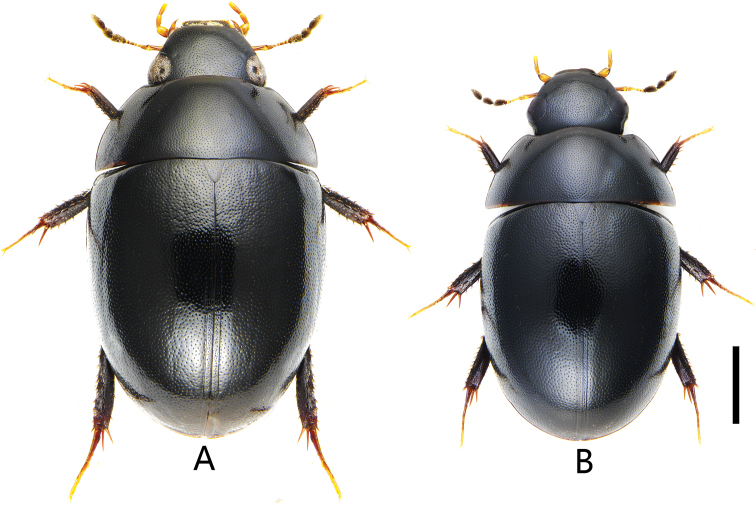
Dorsal view of Chinese *Holocoelostoma***A***Coelostomasulcatum* Pu, 1963 **B***Coelostomastultum* (Walker, 1858). Scale bar: 1.0 mm (**A, B**).

##### Distribution.

China (Guangxi, Jiangxi, Yunnan), Thailand (Jia et al. 2014, 2017). New record for Yunnan.

#### ﻿ Coelostoma (Lachnocoelostoma) hajeki

Taxon classificationAnimaliaColeopteraHydrophilidae

Jia, Aston & Fikáček, 2014

06135430-FE4D-5901-9A27-F3B7D6308E47

##### Material examined.

**China: Hunan**: 30 spec. (SYSU), Yizhang, Mangshan Town, Xiling Village, 24°58'54"N, 112°49'15"E, 642 m, 8.vii.2021, Zu-long Liang leg.

##### Distribution.

China (Guangdong, Guangxi, Hunan). New record for Hunan.

#### ﻿ Coelostoma (Lachnocoelostoma) jaechi

Taxon classificationAnimaliaColeopteraHydrophilidae

Jia, Lin, Chan, Skale & Fikáček, 2017

EA5C86E1-077C-52B7-AEDD-6030359A7407

##### Material examined.

**China: Guangdong**: 1 male (SYSU), Shenzhen City, Dapeng District, Tiantoushan Mountain, Light trap, 22°40'12"N, 114°24'45"E, 208 m, Yang, Jiang, Guo & Ji leg.

##### Distribution.

China (Hong Kong, Guangdong). New record for Guangdong.

#### ﻿ Coelostoma (Lachnocoelostoma) turnai

Taxon classificationAnimaliaColeopteraHydrophilidae

Hebauer, 2006

7395198A-5E8B-50D0-BC43-EC57895F1454

Figure 16A

##### Material examined.

**China: Chongqing**: 4 spec. (SYSU), Jiangjin District, Simianshan Nature Reserve, Dawopu (大窝铺), 20.vi.2014, Jian-yue Qiu & Hao Xu leg.; 1 spec. (SYSU), Jiangjin District, Simianshan Nature Reserve, Er’tai (二台), Hao Xu leg.; **Fujian**: 17 spec. (SYSU), Longyan (龙岩), Mt. Jiangshan(江山), 600 m, 7.iv.2020, Yu-chen Zheng leg.; **Guizhou**: 2 spec. (SYSU), Weng’ang Town, Maolan Nature Reserve, 25°15'08"N, 107°53'56"E, 814 m, 24.vii.2015, Ren-chao Lin & Yu-dan Tang leg.

##### Biology

**(Fig. 16A).** On basis of private communication with the collector Mr. Yu-Chen Zheng (China Agricultural University), the specimens from Fujian were collected on wet rocky ground beside a river at night.

##### Distribution.

Only known from China (Fujian, Guizhou, Hubei, Hunan, Chongqing) (Jia et al. 2014). New record for Fujian, Guizhou, and Chongqing.

#### ﻿ Coelostoma (Lachnocoelostoma) wui

Taxon classificationAnimaliaColeopteraHydrophilidae

Orchymont, 1940

28172BB2-F440-5B0C-AEF2-F7A60D967665

Figures 14A–I , 16I, L

##### Material examined.

**China: Hunan**: 4 spec. (SYSU), Taoyuan County, Wuyunjie, Zhushan Village, 100 m, 15.vi.2019, Hao Xu leg.; **Henan**: 2 spec. (SYSU), Neixiang County, Getiaopa Village (葛条爬村), 630 m, 22.vi.2021, Hao-yi Liu leg.; **Shaanxi**: 1 spec. (SYSU), Xi’an County, Dayu, 12.v.2011, Feng-long Jia leg.; **Shandong**: 3 spec. (HBUM): Feixian, Tashan Forest Farm, 16.–17.v.2007, Feng-yan Wang, Ji-liang Wang & Qi-qi Wu leg.; 2 spec. (HBUM): Pingyi, Dawa Forest Farm, 13–15.v.2007, Feng-yan Wang, Ji-liang Wang & Qiqi Wu leg.; **Shanxi** (山西) : 8 spec. (SYSU), Jincheng, Yangcheng (阳城) County, Manghe (蟒河) National Nature Reserve, 700 m, 27.iv.2016, Zu-qi Mai leg.; **Zhejiang**: 3 spec. (SYSU), Quanzhou City, Jiangshan County, Shuangxikou Town, Laofoyan Village (老佛岩村), 27°55'02.72"N, 119°11'34.47"E, 496 m, high-voltage mercury light trap, 11.viii.2018, Chen & Miao leg.; 7 spec. (SYSU), Lin’an City, Yinlongwu Town, Shibalongtan, 30°08'24.88"N, 118°52'23.56"E, 683 m, 28.iv.2018, Shifting, Cheng & Shen leg.; 2 spec. (SYSU), Jinhua City, Pan’an County, Lingjiangyuan (灵江源), 28°57'39.33"N, 120°38'59.72"E, 750 m, mixed leaf litter, Shifted, 21.V.2018, Puthz, Tang, Cheng & Shuai leg.

##### Additional material examined

**(possibly another undescribed species). China: Guangdong**: 5 spec. (SYSU), Shaoguan City, Chebalin (车八岭) Nature Reserve, 23°14'46"N, 113°33'56"E, 496 m, 28–29.v.2017, Feng-Long Jia, Shi-Shuai Wang & Zu-Long Liang leg.; 24 spec. (SYSU), Shaoguan City, Chebalin (车八岭) Nature Reserve, 24°43'24"N, 114°15'23"E, 400 m, 24.viii.2020, Zu-Long Liang leg.; **Jiangxi**: 91 spec. (SYSU), Longnan, Jiulianshan, 06–vii.2008, Fenglong Jia leg.; **Fujian**: 2 spec. (SYSU), Wuyishan, Sangang, 16–28.v.2004, Cai-Xia Yuan & Jing Li leg.;

##### Biology

**(Figs 15D, 16I, L).** Collected on the ground beside a mountain river in Shanxi, active at night.

##### Remarks.

The specimens we studied contained two different forms of male genitalia (Fig. 14B–I). It may be inferred that the specimens treated by previous authors as *C.wui* Orchymont, 1940 possibly contain two species (see Discussion).

##### Distribution.

China (Hubei, Hunan, Jiangxi, Shaanxi, Shandong, Shanxi, Taiwan, Xinjiang, Zhejiang), Korea. (Hansen 1999; Jia et al. 2014; Liu et al. 2020). First record for Shanxi and Zhejiang.

### ﻿Re-establishment of *Coelostomasulcatum* Pu, 1963 and distribution of *C.stultum* (Walker, 1858)

#### ﻿ Coelostoma (Holocoelostoma) sulcatum

Taxon classificationAnimaliaColeopteraHydrophilidae

Pu, 1963

E759BBB6-D4EB-5EF7-B280-B83DACA7D72B

**Figure 10.**
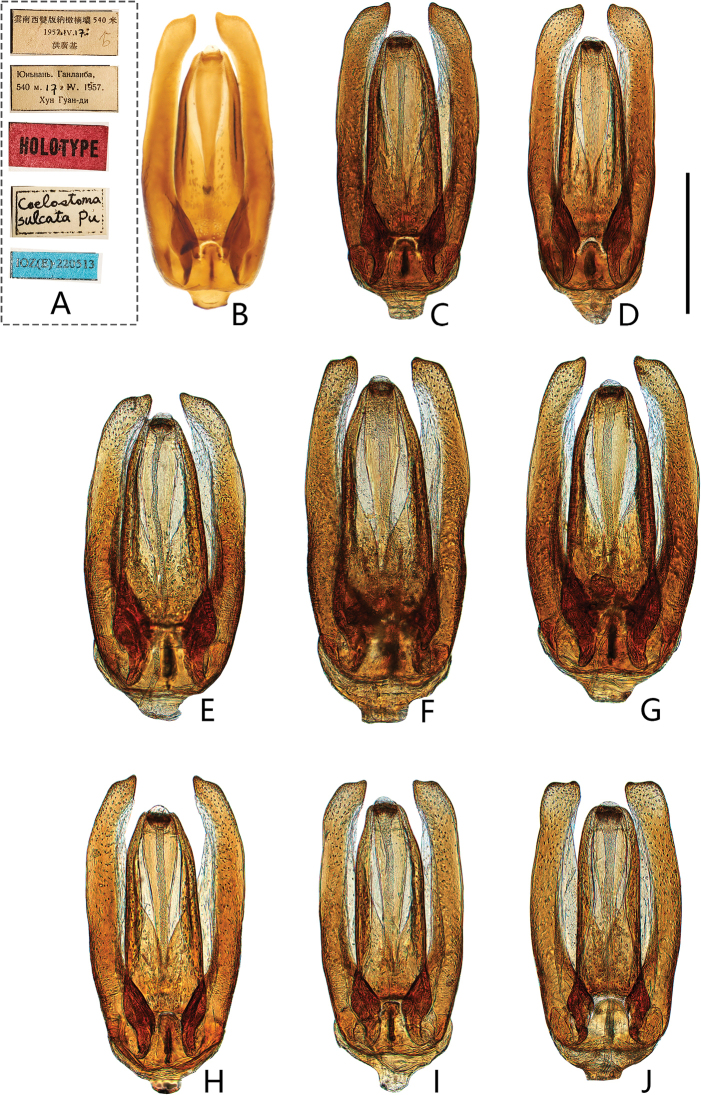
Aedeagus of *Coelostomasulcatum* Pu, 1963 (dorsal view) **A, B** holotype of *C.sulcatum***A** labels **B** aedeagus **C** from Jing’an County (Jiangxi) **D** from Shenzhen City (Guangdong) **E** from Longlin County (Guangxi) **F** from Xishuangbanna (Yunnan) **G** from Xima (昔马) Town (Yunnan) **H** from Tongbiguan Town (Yunnan) **I** from Muotuo County (Xizang) **J** from Macao. Scale bar: 0.5 mm (**A–J**).

Figures 9A , 10A–J , 16B, F, J

Coelostoma sulcatum Pu, 1963: 77. Type locality: Xishuangbanna Dai Autonomous Prefecture, Yunnan, China.
Coelostoma (Holocoelostoma) stultum (Walker, 1858): Jia et al. 2014: 370. Synonym.
Coelostoma (Holocoelostoma) bhutanicum Jayaswal, 1972: Sheth et al. 2020: 21. Possible synonym.

##### Type material examined.

*Coelostomasulcatum*: ***Holotype*** (Fig. 10A, B): male (IZCAS), “Yunnan, Xishuangbanna, Gannanba / 540 m / 1952.IV.17 / Guang-Ji Hong leg. (with Chinese and Russian labels) // *Coelostomasulcata* Pu // HOLOTYPE”; ***Paratype***: male (SYSU), “Jingdong / 1200 m / 26.iv.1957 // A. Monchadskiy leg. (with Chinese and Russian labels) // *Coelostomasulcata* Pu // Paratype”

##### Material examined.

**China: Fujian**: 1 spec. (SYSU), Nanjing County, Hexi Town, in a pond, 13.vii.2010, Feng-long Jia leg.; 2 spec. (SYSU), Ningde District, Mountain behind Ningde Teachers College, 29°01'N, 115°16'E, 315 m, 2.x.2012, Ze-yu Wang leg.; 1 spec. (SYSU), Fu’an District, x.1963, Shan-xiang Lin leg.; **Guangdong**: 1 male (SYSU), Ruyuan County, Longxi, 4–5.x.1964, light trap; 1 spec. (SYSU), Zhuhai, Qi’ao Island, 12.vii.2005, Feng-long Jia leg.; 2 spec. (SYSU), Guangzhou, Shipai, pig farm, 25.vii.1985. Wu Wu leg.; 1 spec. (SYSU), Guangzhou City, Shipai, pig farm, 20.vii.1985. Wu Wu leg.; 1 spec. (SYSU), Guangzhou City, South bank of Zhujiang River, cattle farm, 10.x.1985. Wu Wu leg.; 1 spec. (SYSU), Guangzhou, viii.1938. Zhe-long Pu leg.; 3 spec. (SYSU), Shenzhen City, Inner Lingding Island, 8–12.iv.1998, Peng & Chen leg.; 33 spec. (SYSU), Shenzhen City, Dapeng Peninsula, Getian Village, 22.48157°N 114.52643°E, 12 m, 3.vii.2019, Feng-long Jia & Zu-qi Mai leg.; 1 male (SYSU), Shenzhen City, Dapeng Peninsula, Getian Village, 22°29'25"N, 114°30'59"E, -6 m, 14.xi.2020, Zu-qi Mai, Zhuoyin Jiang & Shu-jiao Jiang leg.; 1 spec (SYSU), Shenzhen City, Dapeng Peninsula, Jin’gui Village, 22°39'35"N, 114°23'10"E, 62 m, 15.v.2019, Wei-cai Xie leg.; 2 spec. (SYSU), Shenzhen City, Dapeng Peninsula, Paiyashan Mountain, 22°37'37"N, 114°26'17"E, 34 m, 5. xi.2018, Lan-bin Xiang leg.; 1 spec (SYSU), Shenzhen, 8–11.viii.2006, Feng-long Jia leg.; 3 spec. (SYSU), Shenzhen City, Pingtouling Mountain, 25.ix.2021, Bao-ping Huang leg.; 1 spec. (SYSU), Danxiashan Mountain, Jinshiyan, pool under a stone wall, 11.vi.2011, Feng-long Jia leg.; 1 male (SYSU), Fengkai County, Heishiding, in a pool, 13.viii.2010, Feng-long Jia leg.; 1 spec. (SYSU), Dinghushan Mountain, 4.vi.1958, Cui-ying Li leg.; **Guangxi**: 1 male (SYSU), Jinxiu County, Luoxiang, 400 m, 16.v.1999, Ming-yuan Gao leg.; 1 male (SYSU), Shangsi County, Hongqi Forestry Centre, 300 m, 29.v.1999, Xing Ke leg.; 4 spec. (SYSU), Nanning City, 22.vi.1958, Zhe-long Pu leg.; 6 spec (SYSU), Nanning City, 19.vi.1977, Zhi-he Huang leg.; Longlin County, Jinzhongshan Mountain, viii.2014, Shan-yi Zhou leg.; **Jiangxi**: 10 spec. (SYSU), Jing’an County, Zaodu (璪都) Town, Nanshan Village (南山村), 315 m, 29°01'N, 115°16'E, 2.viii.2015 Ren-chao Lin & Yu-dan Tang leg.; 4 spec. (SYSU), Jing’an County, Shanzhaolun, Tangli Village (塘里村), 260 m, 29°04'03"N, 115°17'23"E, 3.viii.2015, Ren-chao Lin & Yu-dan Tang leg.; 1 spec. (SYSU), Jing’an County, Jinggangshan, Bijiashan Mountain, 390 m, 26°31'12"N, 114°11'45"E, 22–25.vii.2014, light trap, Chen, Hu, Lv & Yu leg.; 1 spec. (SYSU), Jing’an County, Jinggangshan, Baiyinhu Lake, 800 m, 27.v.2011, Feng-long Jia leg.; **Macao**: 6 spec. (SYSU), Dangzai Mangrove Reserves, First area, 22°8'24"N, 113°33'11"E, 12 m, 16–17.i.2021, on edges of lagoon at night, Feng-long Jia & Zu-qi Mai leg.; 4 spec. (SYSU), Dangzai Mangrove Reserves, First area, 11–12.vii.2018, Feng-long Jia & Wei-cai Xie leg.; 4 spec. (SYSU), Dangzai Mangrove Reserves, First area, 10.x.2020, Feng-long Jia & Wei-cai Xie leg.; 1 spec. (SYSU), Dangzai Mangrove Reserves, First area, 17.vi.2016, Feng-long Jia leg.; 33 spec. (SYSU), Dangzai Mangrove Reserves, First area, 8.iv.2014, Wei-cai Xie & Jin-wei Li leg.; 1 spec. (SYSU), Dangzai Mangrove Reserves, First area, 3.xi.2014, Ren-chao Lin leg.; 1 spec. (SYSU), Coloane, KoloaneAlto (叠石塘), 27.iii.2014, Feng-long Jia leg.; **Taiwan**: 4 spec. (SYSU), Taidung County, Donghe Town, Xinchang (興昌), 25.x.2016, Wen-yi Zhou leg.; **Xizang**: 2 spec., Motuo, Beibeng, 850 m, 25.v.1983, Yinheng Han leg., each with a yellow label “Paratype, *Coelostomaxizangensis*, det. Wu Wu”; 1 male, same data as the former, but with a red label “Holotype, *Coelostomaxizangensis*, det. Wu Wu”; 1 female, same data as the former, but with a label “Allotype, *Coelostomaxizangensis*, det. Wu Wu”; 2 spec., same data as the former, but with a label “*Coelostomaxizangensis*”; 5 spec. (SYSU), Muotuo County, Miri Village, 29°25'06"N, 95°24'23"E, 800 m, 23.vi.2018, Shi-shuai Wang & Zu-long Liang leg.; **Yunnan**: 5 spec. (SYSU), Xishuangbanna Dai Autonomous Prefecture, Botanical Garden, Lake besides Royal Water Lily Hotel, 4–11.iv.2021, Bao-ping Huang leg.; 6 spec. (SYSU), Xishuangbanna Dai Autonomous Prefecture, Botanical Garden, Lake besides Royal Water Lily Hotel, 4–11.iv.2021, Bao-ping Huang leg.; 5 spec. (SYSU), Xishuangbanna Dai Autonomous Prefecture, Botanical Garden, Lake besides Royal Water Lily Hotel, 21.9295°N, 101.2483°E, 500 m, 2.v.2021, Zhuo-yin Jiang, Zhen-ming Yang, Bao-ping Huang & Zu-qi Mai leg.; 1 spec. (SYSU), Xishuangbanna Dai Autonomous Prefecture, Botanical Garden, 21.92262°N, 101.27710°E, 567 m, light trap, 23.v.2011, Ke-qing Song leg.; 2 spec. (SYSU), Xishuangbanna Dai Autonomous Prefecture, Naban Village, 7.i.2004, Li & Tang leg.; 2 spec. (SYSU), Mengla County, Wangtianshu Reserve, light trap, 22.vii.2014, Yun Li leg.; 1 spec. (SYSU), Xishuangbanna Dai Autonomous Prefecture, Gannanba, 500 m, 13.iii.1957, Qiu-zhen Liang leg.; 9 spec. (SYSU), Yingjiang County, Nabang Town, 24.75°N, 97.56°E, 239 m, 27.v.2016, Yu-dan Tang & Rui-juan Zhang leg.; 1 spec. (SYSU), Yingjiang County, Tongbiguan Town, Kaibangya Lake, 24.58°N, 97.67°E, 1289 m, 25.v.2016, Yu-dan Tang & Rui-juan Zhang leg.; 1 spec. (SYSU), Dehong Dai and Jingpo Autonomous Prefecture, Yingjiang County, Xima Town (昔马镇), Hulukou(葫芦口), Xingyun Secondary power station (星云二级电站), 1000 m, vi.2019, light trap, Zhao-yang Tang leg. **Zhejiang**: 1 male (SYSU), Lin’an County, Mt. Tianmushan, 300–400 m, 11–15.vi.2006, Hu & Wang Leg.; 1 spec (SYSU), Mt. W. Tianmushan, 10–21.viii.2004, N.-C. Li Leg.;

##### Diagnosis.

Length 4.5–5.8 mm. Head, pronotum and elytra with similar punctation. Prosternum moderately convex medially, not carinate, without anteromedian process. Elytra slightly or not parallel-sided in the middle, without serial punctures. Mesofemora without dense pubescence, but with punctures bearing strong setae laterally. First abdominal ventrite not carinate, fifth ventrite emarginate and with a row of stout setae apically. ***Aedeagus*** (Fig. 10B–J): 0.9–1.4 mm long. Median lobe widest basally, almost parallel from base to apical fourth, then distinctly narrowed apically (materials from Macao slightly narrowed); gonopore situated apically. Parameres longer than median lobe, outer face slightly curved or sinuate medially and strongly curved inwards apically.

##### Biology

**(Figs 15G, H, 16B, F, J).** This species can be found in various of aquatic environments. It can be collected on wet ground near rivers, streams or natural lakes. It also occurs at some artificial environments, such as on sandy gutters with very shallow flowing waters in Shenzhen (Fig. 16B), on the edges of an artificial lake (Fig. 15G) and also lives with *Coelostomaphallicum* Orchymont, 1940 in Xishuangbanna. It also occurs on the muddy edges of a brackish lagoon in a mangrove reserve in Macao (Fig. 15H). Adults are active at night and sometimes attracted by light.

##### Remarks.

Jia et al. (2014) thought this species was a synonym of *Coelostomastultum* Walker. Liu et al. (2020) and Sheth et al. (2020) stated that it is a different species from *C.stultum* Walker after they studied a paratype of *C.stultum*, and considered as a likely synonym of *Coelostomabhutanicum* Jayaswal, 1972 (Sheth et al. 2020). Here, we recovered the status of *C.sulcatum* Pu as a valid species.

*Coelostomasulcatum* Pu, 1963 is morphologically variable in shape of aedeagus, especially in parameres. Compared with the original description (Jayaswal 1972) and photos of *C.bhutanicum* from india (Sheth et al. 2020), *C.bhutanicum* is very similar to *C.sulcatum* especially in aedeagus, which outer face of median lobe is slightly narrowing or subparalleling from base to apical fourth and distinctly narrowed subapically (Fig. 10B–I). This indicates *C.bhutanicum* and *C.sulcatum* possibly refer to the same species.

Liu et al. (2020) reported *C.bhutanicum* Jayaswal, 1972 from Taiwan. In his illustration of aedeagus, median lobe is of almost same width throughout, only slightly narrowed apically (Liu et al. 2020: fig. 2E). This character is inconsistent with the median lobe of *C.bhutanicum* drawn by Jayaswal (1972), but very closed to *C.bhutanicum* from Japan (Watanabe and Minoshima 2020) and *C.sulcatum* from Macao (Fig. 10J). This character has not been found in other specimens of *C.sulcatum* and *C.bhutanicum*. Hence, it is possible that the specimens with this character represent another undescribed species or just intraspecific variation of *C.sulcatum*. However, solving the problem of synonymization of *C.bhutanicum* and *C.sulcatum* is not easy until the type of *C.bhutanicum* can be examined. Hence, we prefer to treat specimens from China as *C.sulcatum* and not treat *C.bhutanicum* in the key to species of Chinese *Coelostoma* at present.

##### Distribution.

China (Fujian, Guangdong, Guangxi, Jiangxi, Macao, Taiwan, Yunnan, Xizang, Zhejiang).

#### ﻿ Coelostoma (Holocoelostoma) stultum

Taxon classificationAnimaliaColeopteraHydrophilidae

(Walker, 1858)

1D56FE05-3C79-5952-B481-8C15736051AF

Figures 9B , 11A–F

##### Note.

For complete synonymy, see Hansen (1999).

##### Material examined.

**China: Guangdong**: 3 spec. (SYSU), Sihui District, Dasha Town, 5.vi.1998, Feng-long Jia leg.; 1 male (SYSU), Huaxian County, Dapuling, 26.viii.1983, Zhi-he Huang leg.; 2 spec. (SYSU), Lianjiang District, 25.ix.1985, Wu Wu leg.; 1 male (SYSU), Xinhui District, viii.2001, Xiao-li Tong leg.; **Guangxi**: 45 spec. (SYSU), Yangshuo (阳朔), viii.1984, Shou-jian Chen leg.; 6 spec. (SYSU), Shiwandashan Forest Park, 267 m, light trap, 9.vii.2011, Ke-qing Song leg.; **Hainan**: 1 male (SYSU), Lingshui, Diaoluoshan Mountain, Xiaomei (小妹), 29.xi.1963, Bai-ge Chen leg.; **Hubei**: 1 male (SYSU), Wuhan City, Wuhan Botanical Garden, 31.viii.2020, Zi-hao Shen leg.; **Hunan**: 2 spec. (SYSU), Yizhang (宜章), 16.iii.1941, Zhe-long Pu leg.; **Jiangxi**: 4 spec. (SYSU), Jing’an County, Guanyinyan (观音岩), 29°01'48"N, 115°25'00"E, 195 m, 1.viii.2015, Ren-chao Lin & Yu-dan Tang leg.; 3 spec. (SYSU), Jiujiang District, Duchang County, Linshan Village Committee, 15–20.viii.2010, Yan Mei leg.;**Yunnan**: 5 spec. (SYSU), Mengla Nature Reserve, 4–5.viii.2007, Jia-hui Li leg.; **Zhejiang**: 1 male (SYSU), Quzhou City, Kecheng District, 29.0063°N, 118.8063°E, 112 m, 15.iii.2020, Zhuo-yin Jiang leg.; 1 male (SYSU), Quzhou City, Kecheng District, Wangdu Village, 28.9875°N, 118.6898°E, 102 m, 7.iii.2020, Zhuo-yin Jiang leg.; 7 spec. (SYSU), Kaihua County, Zawanhua (砸碗花) Wetland Park, 11.iii.2020, Zhuo-yin Jiang leg.

**Figure 11.**
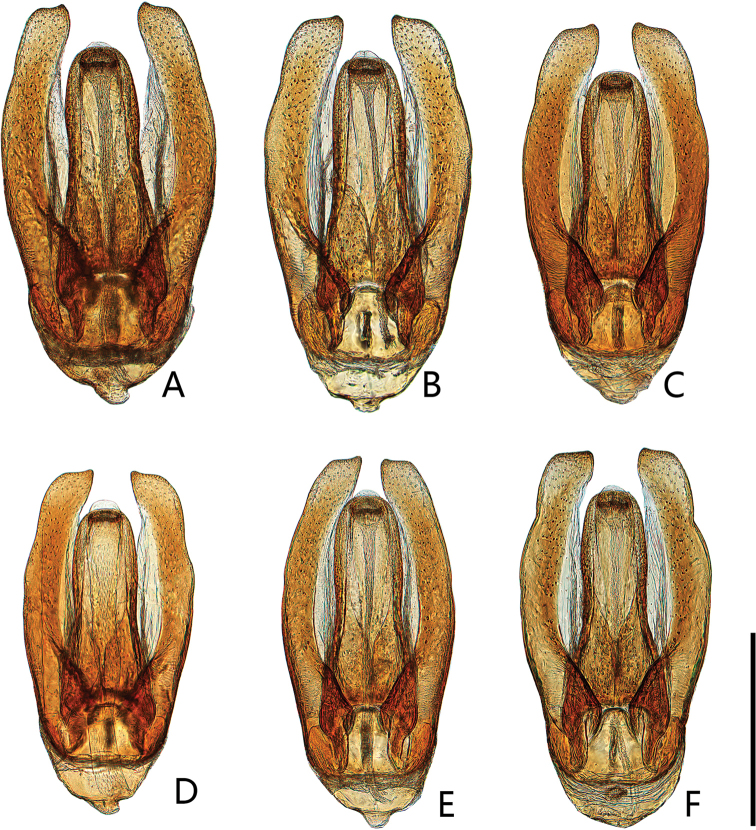
Aedeagus of *Coelostomastultum* (Walker, 1858) (dorsal view) **A** from Huaxian County (Guangdong) **B** from Sihui District (Guangdong) **C** from Mt. Shiwandashan (Guangxi) **D** from Yangshuo (Guangxi) **E** from Yizhang (Hunan) **F** from Mt. Diaoluoshan (Hainan). Scale bar: 0.5 mm (**A–F**).

##### Additional material examined.

**Cambodia**: 17 spec. (SYSU), Boeng, Kampong Thom Pro., Khleng, light trap beside a rice field, 13.viii.2017, Zu-qi Mai leg.

##### Diagnosis.

Length 4.3–5.2 mm. similar to *C.sulcatum* in morphological characters. ***Aedeagus*** (Fig. 11A–F): 0.8–1.0 mm long. Median lobe widest basally, distinctly narrowed medially, then slightly widened at apical third; gonopore situated apically. Parameres longer than median lobe, outer face continually curved and strongly curved inwards apically.

**Figure 12.**
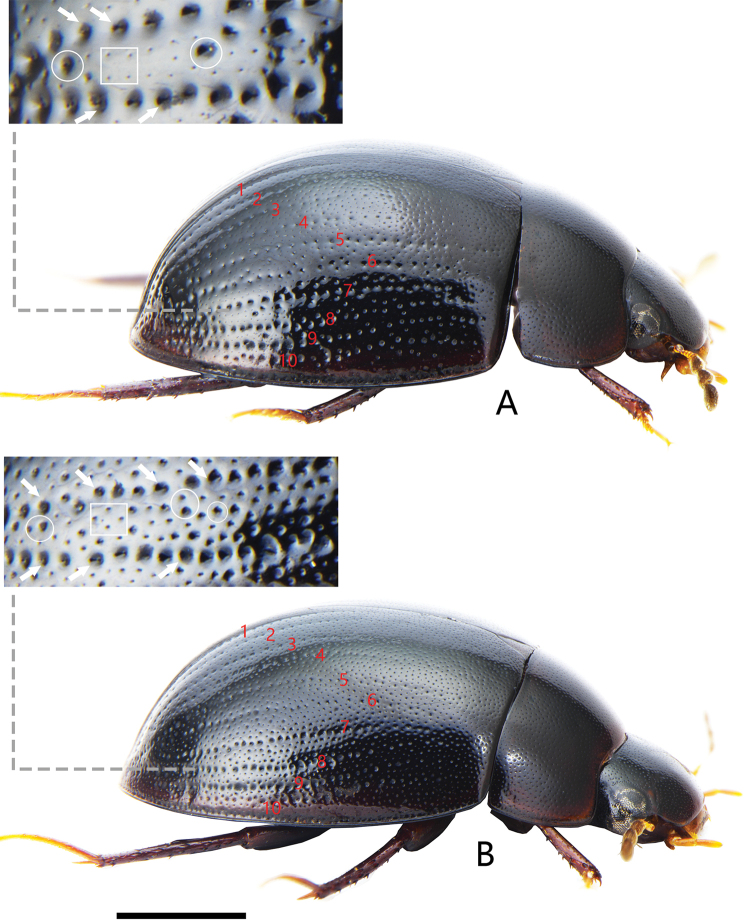
Lateral view of *Coelostoma* spp. (the red figures indicate the serial number of elytral serial punctures; the white arrows indicate the punctures of series; the circles indicate the coarser punctures; the squares indicate the finer punctures) **A***Coelostomadactylopunctum* sp. nov. **B***Coelostomafortunum* sp. nov. Scale bar: 1 mm (**A, B**).

##### Biology.

This species has the similar habitations of *C.sulcatum* Pu, 1963.

**Figure 13.**
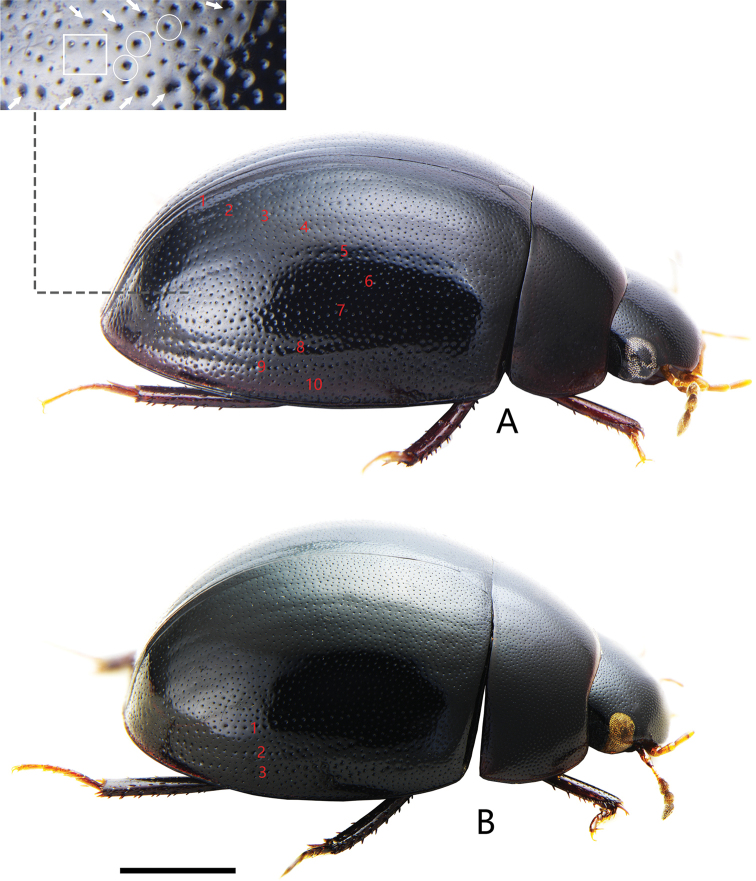
Lateral view of *Coelostoma* spp. (the red figures indicate the serial number of elytral serial punctures; the white arrows indicate the punctures of series; the square indicates the elytral ground punctures) **A***Coelostomapseudomartensi* sp. nov. **B***Coelostomagentilii* Jia, Aston & Fikáček, 2014. Scale bar: 1 mm (**A, B**).

##### Occurrence in China.

This species was reported widely distributed in China (Chongqing, Fujian, Guangxi, Guangdong, Jiangxi, Hainan, Hunan, Hong Kong, Shandong, Sichuan, Taiwan, Yunnan, Xizang) (Jia et al. 2014, 2017; Liu et al. 2020; Przewoźny 2021). However, some records should be based on misidentification. We examined all the specimens assigned as *C.stultum* in SYSU. After excluded the specimens of *C.sulcatum*, the distribution of *C.stultum* in China is confirmed: Guangdong, Guangxi, Jiangxi, Hainan, Hubei, Hunan, Taiwan, Yunnan, Zhejiang.

**Figure 14.**
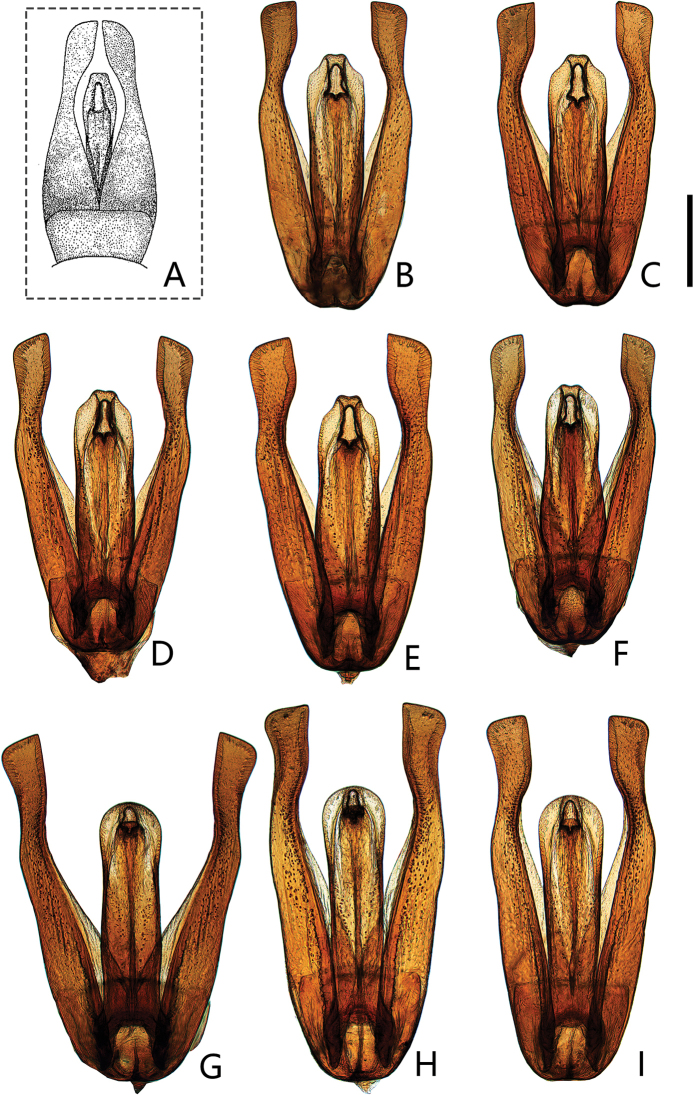
Aedeagus of *Coelostomawui* Orchymont, 1940 (dorsal view) **A** illustration by Orchymont (1940)**B** from Pingyi County (Shandong) **C** from Getiaopa (葛条爬) Village (Henan) **D** from Hanzhong (Shaanxi) **E** from Taoyuan County (Hunan) **F** from Quanzhou City (Zhejiang) **G** from Wuyishan (Fujian) **H** from Mt. Jiulianshan (Jiangxi) **I** from Mt. Chebaling (Guangdong). Scale bar: 0.5 mm.

**Figure 15.**
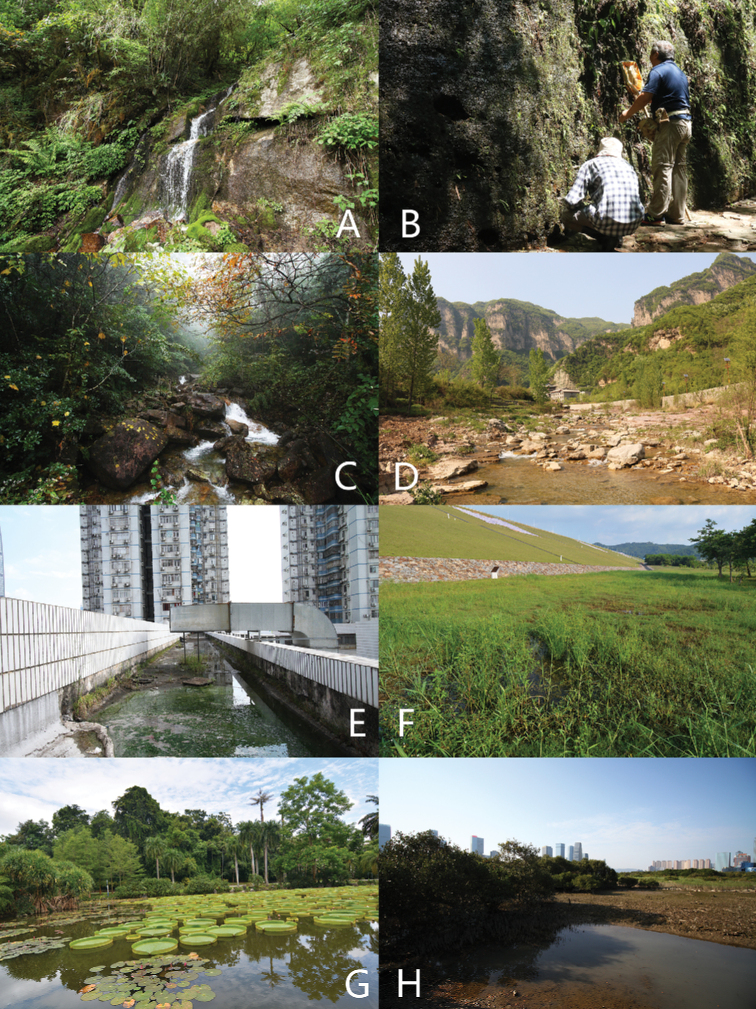
Habitats of Chinese *Coelostoma***A** stone walls with running waters in forest (Yunnan) **B** wet stone walls with moss (Guangdong) **C** mountain stream in forest (Guangdong) **D** mountain river with rocky edges (Shanxi) **E** wastewater in city downtown (Guangdong) **F** lowland marshes with vegetation (Guangdong) **G** artificial lake (Yunnan) **H** brackish lagoon in mangrove reserve (Macao).

**Figure 16.**
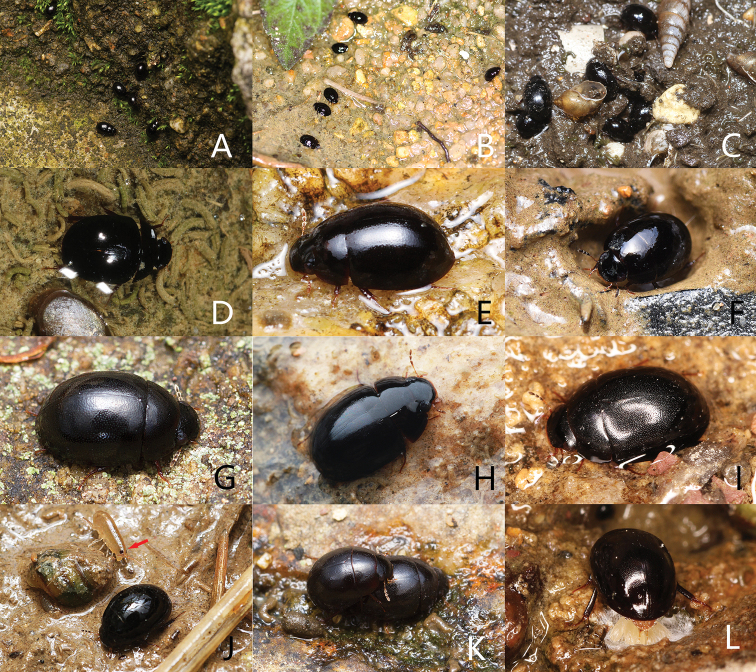
Habitats of Chinese *Coelostoma* spp. **A***C.turnai* Hebauer, 2006 active at wet rocky ground beside a river at night (Fujian) **B***C.sulcatum* Pu, 1963 on sandy gutterway with shallow flowing waters at night (Guangdong) **C***C.phallicum* Orchymont, 1940 hided under a brick beside the wastewater during the day (Guangdong) **D***C.phallicum* Orchymont, 1940 dived under water and fed on algal mats at night (Guangdong) **E***C.bifidum* Jia, Aston & Fikáček on wet stone wall at night (Guangdong) **F***C.sulcatum* Pu, 1963 on muddy edge of artificial lake at night (Yunnan) **G***C.nankunshanense* sp. nov. on a stone in the middle of a forest stream at night (Guangdong) **H***C.surkhetensis* Hebauer, 2002 in a wet stone on the edges of a forest stream at night (Xizang) **I***C.wui* Orchymont, 1940 on the edges of a mountain river at night (Shanxi) **J***C.sulcatum* Pu, 1963 on muddy edges of brackish lagoon at night, with a marine Amphipoda beside it (Macao) **K***C.nankunshanense* sp. nov. mating at night (Guangdong) **L***C.wui* Orchymont, 1940 ovipositing eggs on wet land at night (Shanxi).

### ﻿A key to species of Chinese *Coelostoma*

This key is modified based on Jia et al. (2014, 2017) and Liu et al. (2020). *Coelostomataiwanense* Liu, Hu & Fikáček, 2020 from Taiwan is based on the original description.

**Table:** 

1	Mesofemora densely pubescent except at extreme apex (Jia et al. 2014: fig. 9). (Subgenus Lachnocoelostoma)	**2**
–	Mesofemora not pubescent, glabrous, more or less coarsely punctate and sparsely covered by short setae (Jia et al. 2014: figs 11, 13)	**24**
2	Elytra with distinct serial punctures laterally (Figs 12A, B, 13A, B)	**3**
–	Elytra without serial punctures laterally	**6**
3	Elytra serial punctures only visible laterally, without serial punctures on disc (Fig. 13B). Gonopore situated at midlength of median lobe (Jia et al. 2014: fig. 25)	***C.gentilii* Jia, Aston & Fikáček, 2014**
–	Elytra series punctures visible on disc (Figs 12A, B, 13A). Gonopore situated subapically (Figs 2D, E, 3D, E, 7D, E)	**4**
4	Elytron with 10 serial punctures, somewhat difficult to separate from the ground punctures in anterior half of elytron (Fig. 13A). Inner face of paramere straight, not curved inwards apically; median lobe rounded apically, not truncate or emarginate apically (Fig. 7D, E)	***C.pseudomartensi* sp. nov.**
–	Elytron with 10 distinct serial punctures (Fig. 12A, B). Inner face of paramere curved inwards apically; apex of median lobe truncate or emarginate (Figs 2D, E, 3D, E)	**5**
5	Intervals between series with two sizes of punctures, the small punctures much finer and shallower than the big punctures, big punctures almost as coarse as those of the series (Fig. 12A). First ventrite with complete median carina. Median lobe emarginate apically, outer face nearly parallel-sided from basal to middle, then gradually narrowing towards apex; gonopore distinctly wider than long; paramere gradually expanded from anterior fourth to apex, broadly truncate apically (Fig. 2D, E)	***C.dactylopunctum* sp. nov.**
–	Intervals between series with two sizes of punctures, all finer than those of the series, the small punctures finer and shallower than the big punctures but not extremely so (Fig. 12B). First ventrite with median carina on basal two-thirds. Median lobe truncate apically, not emarginate, outer face nearly parallel-sided throughout; gonopore rounded, almost as wide as long; apex of paramere pointed (Fig. 3D, E)	***C.fortunum* sp. nov.**
6	Body size < 4.0 mm. Pronotum with much finer and sparser punctation than on elytra (Jia et al. 2014: fig. 7). Gonopore situated almost at midlength, median lobe with distinct lateral projections (Jia et al. 2014: fig. 23)	***C.hongkongense* Jia, Aston & Fikáček, 2014**
–	Body size > 4.0 mm. Pronotum with punctation at most slightly finer and sparser than punctation on elytra. Median lobe of the aedeagus without subapical lateral projections	**7**
7	Median lobe of aedeagus trilobate apically	**8**
–	Median lobe of aedeagus not emarginate to deeply emarginate apically	**10**
8	Aedeagus narrowly elongate; Median lobe not wider than paramere (Jia et al. 2014: fig. 26)	***C.phallicum* Orchymont, 1940**
–	Aedeagus relatively wider; median lobe wider than paramere	**9**
9	Aedeagus large (ca. 1.1 mm long), median lobe strongly sclerotized, highly modified, saddle-shaped in lateral view, rather shorter than parameres; parameres rather broadened subapically inwards (Jia et al. 2017: figs 1–4)	***C.tangliangi* Jia, Lin, Chan, Skale & Fikáček, 2017**
–	Aedeagus small (ca. 0.6 mm long), weakly sclerotized, median lobe plain, only slightly bent in lateral view, not so shorter than parameres; parameres no so broadened subapically inwards (Jia et al. 2017: figs 5–8)	***C.horni* (Régimbart, 1902)**
10	Aedeagus large (> 1.5 mm long), parameres largely overlapping apex of median lobe	**11**
–	Aedeagus smaller (< 1.5 mm long), parameres only slightly longer than median lobe	**14**
11	Apex of the median lobe widely rounded or slightly emarginated, parameres broadly widened apically (Fig. 14A–I)	***C.wui* Orchymont, 1940**
–	Apex of the median lobe narrowly rounded or angulate	**12**
12	Median lobe widest in the middle, with a small rounded finger like apex; parameres broadly widened apically (Fig. 5A–C)	***C.mixtum* sp. nov.**
–	Median lobe nearly parallel-sided in the middle, apex pointed or augulate; parameres not distinctly widened apically	**13**
13	Apex of median lobe widely augulate; parameres weakly narrowing in apical third, slightly bent inward (Liu et al. 2020: fig. 1C)	***C.taiwanense* Liu, Hu & Fikáček, 2020**
–	Median lobe strongly narrowing near apex, apex with a sharp prominent hook-shaped tooth ventrally; parameres relatively slender, with inner face almost straight (Fig. 5D, E)	***C.vagum* Orchymont, 1940**
14	Median lobe of aedeagus distinctly emarginate apically; gonopore situated basally or slightly before the midlength of the median lobe	**15**
–	Median lobe of aedeagus not or slightly emarginate; gonopore situated subapically to apically	**19**
15	Median lobe bottle-shaped, strongly broadened basally; gonopore situated basally	**16**
–	Median lobe widest in the middle; gonopore situated slightly above the midlength of the median lobe	**18**
16	Outer face of parameres convex basally; median lobe strongly widened basally, gonopore extremely transverse	**17**
–	Outer face of parameres nearly straight basally; basal portion of median lobe moderately widened, gonopore transverse round (Jia et al. 2014: fig. 22)	***C.hajeki* Jia, Aston & Fikáček, 2014**
17	Median strongly broadened basally. Outer face of parameres distinctly incised subapically (Fig. 6D–F)	***C.nankunshanense* sp. nov.**
–	Median lobe not so broadened basally. Outer face of parameres slightly curved subapically (Jia et al. 2014: fig. 21)	***C.turnai* Hebauer, 2006**
18	Median lobe deeply emarginate apically; apex of paramere widened, sharply protruding inwards (Jia et al. 2014: fig. 20)	***C.bifidum* Jia, Aston & Fikáček, 2014**
–	Median lobe shallowly emarginate apically; paramere not widended apically, obtusely truncate at apex (Jia et al. 2014: fig. 24)	***C.coomani* Orchymont, 1932**
19	Median lobe of aedeagus not emarginate apically	**20**
–	Median lobe of aedeagus slightly emarginate or truncate apically	**21**
20	Median lobe with a distinct subapical tooth and a lateral ridge subapically (Jia et al. 2019: figs 2, 3)	***C.jaculum* Jia, Angus & Bian, 2019**
–	Median lobe with a rounded apex (Jia et al. 2019: fig. 1)	***C.phototropicum* Jia, Angus & Bian, 2019**
21	Parameres obliquely truncate inwards apically (Jia et al. 2017: fig. 9)	***C.huangi* Jia, Aston & Fikáèek, 2014**
–	Inner face of parameres rounded or augulate apically	**22**
22	Parameres strongly expanded apically, apex of paramere distinctly wider than apex of median lobe	**23**
–	Parameres not expanded apically, apex of paramere narrower than apex of median lobe (Fig. 8D, E)	***C.surkhetensis* Hebauer, 2002**
23	Size larger than 5.0 mm. Median lobe widest at midlength (Fig. 1D, E)	***C.bannanicum* sp. nov.**
–	Size smaller than 5.0 mm. Median lobe widest at apical third (Jia et al. 2017: fig. 11)	***C.jaechi* Jia, Lin, Chan, Skale & Fikáček, 2017**
24	Fifth abdominal ventrite slightly emarginate posteromesally, bearing strong setae mesally (Jia et al. 2014: fig. 30) (subgenus Holocoelostoma)	**25**
–	Posterior margin of the fifth abdominal ventrite entire, not emarginate in the middle (Jia et al. 2014: fig. 32) (subgenus Coelostoma s. str.)	**26**
25	Median lobe almost parallel at basal third-fourth, apical fourth distinctly narrowed subapically; outer face of parameres more or less parallel, only slightly curved medially (Fig. 10B–J)	***C.sulcatum* Pu, 1963**
–	Median lobe distinctly narrowed medially, then slightly widened at apical third; outer face of parameres broadened medially (Fig. 11A–F)	***C.stultum* (Walker, 1858)**
26	Aedeagus slender, median lobe gradually attenuate toward apex, sharpened apically. Parameres strongly narrowed from apical fifth to apex, pointed apically (Jia et al. 2014: fig. 19)	***C.orbiculare* (Fabricius, 1775)**
–	Aedeagus robust, median lobe and parameres not strongly narrowing apically	**27**
27	Posterior femora broad to almost oval in form. Median lobe of aedeagus broad and short, parameres slender (Jia et al. 2014: fig. 16)	***C.vitalisi* Orchymont, 1923**
–	Posterior femora not broadened, aedeagus not as above	**28**
28	Body length 4.1–4.2 mm. Mesofemora finely and sparsely punctate. Median lobe of aedeagus strongly broadened at basal half, abruptly narrowed mesally, and almost parallel-sided in apical half, gonopore subtriangular, situated ca. at midlength of the median lobe (Jia et al. 2014: figs 17, 18)	***C.vividum* Orchymont, 1936**
–	Body length 4.7–5.4 mm. Mesofemora with coarse and dense punctation. Median lobe of aedeagus not so broadened basally and not so extremely narrow from middle to apex, gonopore apical or subapical	**29**
29	Median lobe of the aedeagus bottle-shaped, with broad base and abruptly narrowed and gradually slightly narrowed toward apex, gonopore in shape of number 8 (Jia et al. 2014: fig. 15)	***C.fallaciosum* Orchymont, 1936**
–	Median lobe of aedeagus gradually narrowed from base to apex, not abruptly narrowed, gonopore rhomboid in shape (in Jia et al. 2014: figs 12–14)	***C.subditum* Orchymont, 1936**

## ﻿Discussion

### ﻿Species excluded from Chinese fauna

Orchymont (1925) reported *Coelostomatranscaspicum* Reitter from China based on a series of specimens from Kiau-Tschau (Shandong) without examining the male genitalia (Balfour-Browne 1951). Later, he treated these specimens as *Coelostomawui* without description (Orchymont 1935). When Orchymont (1940) described *C.wui*Orchymont 1940 as a new species, he assigned a male of the specimens from Kiau-Tschau as the holotype. Balfour-Browne (1951) also doubted the reliability of *C.transcaspicum* recorded by Orchymont in China. There is no doubt that the record of *C.transcaspicum* in China by Orchymont (1925) is based on misidentification because he assigned the specimens identified by him as *C.transcaspicum* Reitter in 1925 as *C.wui* Orchymont. Other reports on *C.transcaspicum* Reitter from China (Hansen 1999; Jia et al. 2019; Przewoźny 2021) are based on Orchymont (1925) report without any further examination to materials. We hence remove this species from the Chinese fauna. The records from other areas of Oriental realm are also dubious (Balfour-Browne 1951).

### ﻿The status of Coelostoma (Lachnocoelostoma) wui Orchymont, 1940

Orchymont (1940) described *Coelostomawui* Orchymont, 1940 from Kiau-Tschau (Shandong) and Chin-Kiang (Xinjiang). Jia et al. (2014, 2017) reported *C.wui* from Fujian, Henan, Hubei, Hunan, Jiangxi and Shaanxi. Liu et al. (2020) reported *C.wui* from Taiwan. Hence it is widely distributed in China based on the reports above.

Jia et al. (2014) and Liu et al. (2020) illustrated the male genitalia of *C.wui*, which both clearly show the widely rounded apex of median lobe. This character is different from the aedeagus of *C.wui* drawn by Orchymont (1940) (Fig. 14A), but it has been neglected maybe because Orchymont’s drawing was based on a dehydrated aedeagus. However, after we dissected more specimens assigned to *C.wui* Orchymont, 1940 from different locations, we found there are two different forms of aedeagus among these specimens, one of which has the median lobe corresponding with the illustrations by Jia et al. (2014) and Liu et al. (2020) (Fig. 14G–I), while the other form of median lobe is slightly emarginate apically and obliquely truncate subapically (Fig. 14B–F). This later one is more in conformity with Orchymont’s drawing of the apex of the median lobe, but its median lobe does not abruptly widen subapically as Orchymont’s drawing.

All specimens with an emarginate apex were found from the north of Nanling Mountains (Henan, Hubei, Hunan, Shandong, Shaanxi, Shanxi and Zhejiang), one of which (Pingyi County) (Fig. 14B) is close to the type locality of *C.wui*. Conversely, the specimens with a rounded apex of median lobe were only collected from the south of Nanling Mountains (Mt. Chebaling in Guangdong, Mt. Jiulianshan in Jiangxi and Wuyishan area in Fujian). This indicates the possibility that the specimens with a rounded apex of median lobe and the specimens with an emarginate apex of median lobe each maybe represents different species. The specimens collected from the south of Nanling Mountains, with a widely rounded apex of median lobe possibly represents another undescribed species (Fig. 14G–I). We prefer to treat all of specimens as *C.wui* here until the type of *C.wui* can be examined in order to prevent new synonymy.

### ﻿The diversity and habitats of Chinese *Coelostoma* Brullé

The genus *Coelostoma* is a typical tropical group, only several species occurring in temperate region (Hansen 1999; Jia et al. 2014; Fikáček et al. 2019). In China, 90% species are only known from south of the Qinling Mountain-Huaihe River line, which is considered as the boundary of southern and northern China. The species diversity of the genus in China is high in Yunnan, Guangxi, Guangdong, Jiangxi Province and southeast part of Xizang where there is a part of Oriental realm (Jia et al. 2014, 2017, 2019), probably because of the warm and moist climate and the mountain terrain conditions.

Currently, 30 species of *Coelostoma* are known from China, of which two species are assigned to the subgenus Holocoelostoma, five species to *Coelostoma* (s. str.), and 23 species to *Lachnocoelostoma*. Compared with the fauna of other regions, we can come to following conclusions: (1) the Chinese fauna has a large species diversity of *Lachnocoelostoma* and nearly half of them seem to be very local and are likely endemic (Jia et al. 2014, 2017, 2019; Liu et al. 2020). (2) The diversity of *Coelostoma* (s. str.) and *Holocoelostoma* is comparatively low in China, all of them widespread species in Oriental realm (Jia et al. 2014, 2017; Liu et al. 2020; Sheth et al. 2020). This phenomenon also happens in some other Oriental fauna (Sheth et al. 2020). Sheth et al. (2020) considered this pattern of Oriental *Coelostoma* depends on their habitat preferences: Oriental species of *Coelostoma* (s. str.) and *Holocoelostoma* occur in lowland standing waters, while *Lachnocoelostoma* has a much wider spectrum of habitats, including running waters (Sheth et al. 2020).

Previous studies on aquatic beetles indicate that species occurring in standing water tend to have larger ranges than species in running waters (e.g., Ribera and Vogler 2000; Liang et al. 2021). The assumption by Sheth et al. (2020) might explain why *Lachnocoelostoma* tends to have a larger diversity than *Coelostoma* (s. str.) in China. South China (Yunnan, Guangxi, Guangdong, Jiangxi, Taiwan and southeast part of Xizang) is a mountainous area where is fit to live for most of *Lachnocoelostoma* species. Some endemic species of *Lachnocoelostoma* only inhabit wet stone walls and edges of clean running water in mountainous areas (Fig. 15A–D), such as *C.bifidum* Jia, Aston & Fikáček, *C.hajeki* Jia, Aston & Fikáček, and *C.huangi* Jia, Aston & Fikáèek (Jia et al. 2014). They were rarely collected in flatlands. In contrast, some widespread species of *Coelostoma* (s. str.) mostly occur in lowland standing water (Fig. 15F) (for example *C.fallaciosum* Orchymont). A few species of *Lachnocoelostoma* are widespread in different kinds of aquatic environments, even in cities. For example, Liu (2021) reported *C.phallicum* Orchymont inhabits water ditches in city streets of Taiwan. We also found *C.phallicum* occurring in wastewater, which leaked from the drain pipes under a tall residential building in downtown Shenzhen (Guangdong) (Figs 15E, 16C). In the wild, *C.phallicum* usually inhabits edges of mountain streams, rice fields and lowland ponds (Fig. 15G). Some *Holocoelostoma* can also be found in both standing and running waters (Fig. 16B), even in brackish water (Figs 15H, 16J) (for example *C.sulcatum* Pu). The species of *Holocoelostoma* are usually more widespread than species of *Lachnocoelostoma* and *Coelostoma* (s. str.), but with low diversity.

**Figure 17.**
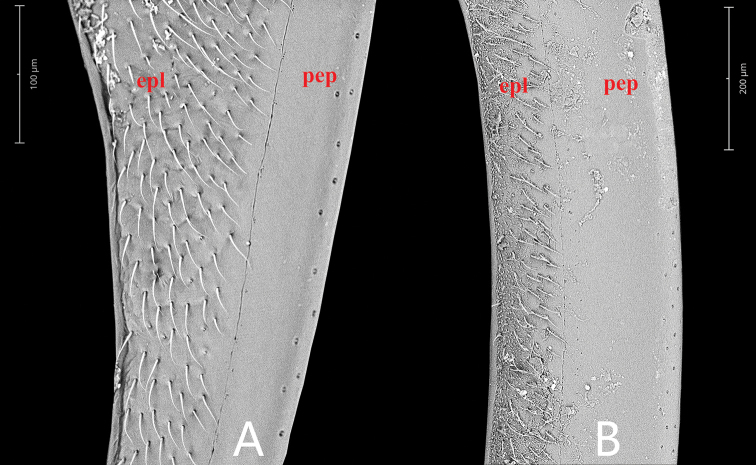
SEM micrographs of epipleuron (epl) and pseudepipleuron (pep) at the metacoxa level **A***Coelostomadactylopunctum* sp. nov. **B***Dactylosternumlatum* (Sharp, 1873).

### ﻿The characters of Chinese *Coelostoma* Brullé

Hansen (1991) provided the following characters as diagnostic characteristics for *Coelostoma*: (1) the first segment of hind tarsi distinctly longer than second, (2) the antennal club loosely segmented, (3) tarsi without fringe of swimming hairs, (4) elytra without serial punctures. However, the third and fourth characteristics show considerable variation among known Oriental Coelostoma species, especially in subgenus Lachnocoelostoma. For example, *C.thienemanni* Orchymont, 1932 with distinct dorsal swimming hairs on meso- and metatarsi (Fikáček et al. 2019); *C.martensi* Hebauer, 2002 and *C.gentilii* Jia, Aston & Fikáček, 2014 with serial punctures on elytra sublaterally; three new species described here, *C.dactylopunctum* sp. nov., *C.fortunum* sp. nov. and *C.pseudomartensi* sp. nov., with distinct serial punctures on the disc and lateral portion of the elytra (Figs 12A, B, 13B), which seems not to support these species as members of *Coelostoma*. These three species may be recognized as members of *Dactylosternum* based on this character. However, they are all aquatic (except biology of *C.fortunum* sp. nov. remains unknown), and extremely similar to all other species of *Coelostoma*. So, they are hence considered as members of *Coelostoma*. The terrestrial genus *Dactylosternum* seems to serve as a “dustbin” for coelostomatine species lacking any apparent generic characters (e.g., Fikáček 2010). Many species assigned to *Dactylosternum* lack biological information as do some unusual *Coelostoma* species (private communication from Fikáček). As a result, we summarized the characteristics combined for distinguishing *Coelostoma* from *Dactylosternum* (1) aquatic or amphibious, (2) antennal club loosely segmented, (3) elytra with or without serial punctures, (4) elytral margin not explanate (except some species slightly explanate), (5) epipleuron wider than pseudepipleuron at the metacoxa level (Fig. 17A) (epipleuron distinctly narrower than pseudepipleuron at the metacoxa level in *Dactylosternum*; Fig. 17B). These characters allow a reliable identification of all Chinese species.

## Supplementary Material

XML Treatment for Coelostoma (Lachnocoelostoma) bannanicum

XML Treatment for Coelostoma (Lachnocoelostoma) dactylopunctum

XML Treatment for Coelostoma (Lachnocoelostoma) fortunum

XML Treatment for Coelostoma (Lachnocoelostoma) mixtum

XML Treatment for Coelostoma (Lachnocoelostoma) nankunshanense

XML Treatment for Coelostoma (Lachnocoelostoma) pseudomartensi

XML Treatment for Coelostoma (Lachnocoelostoma) surkhetensis

XML Treatment for Coelostoma (Lachnocoelostoma) huangi

XML Treatment for Coelostoma (Lachnocoelostoma) hajeki

XML Treatment for Coelostoma (Lachnocoelostoma) jaechi

XML Treatment for Coelostoma (Lachnocoelostoma) turnai

XML Treatment for Coelostoma (Lachnocoelostoma) wui

XML Treatment for Coelostoma (Holocoelostoma) sulcatum

XML Treatment for Coelostoma (Holocoelostoma) stultum
